# Age-dependent brain responses to mechanical stress determine resilience in a chronic lymphatic drainage impairment model

**DOI:** 10.1172/JCI182555

**Published:** 2025-07-15

**Authors:** Zachary Gursky, Zohaib Nisar Khan, Sunil Koundal, Ankita Bhardwaj, Joaquin Caceres Melgarejo, Kaiming Xu, Xinan Chen, Hung-Mo Lin, Xianfeng Gu, Hedok Lee, Jonathan Kipnis, Yoav Dori, Allen Tannenbaum, Laura Santambrogio, Helene Benveniste

**Affiliations:** 1 Department of Anesthesiology, Yale School of Medicine, New Haven, Connecticut, USA.; 2 Department of Radiation Oncology, Weill Cornell Medicine, New York, New York, USA.; 3 Department of Medical Physics, Memorial Sloan Kettering Cancer Center, New York, New York, USA.; 4 Departments of Computer Science and Applied Mathematics & Statistics, Stony Brook University, Stony Brook, New York, USA.; 5 Brain Immunology and Glia (BIG) Center, Department of Pathology and Immunology, Washington University School of Medicine, St. Louis, Missouri, USA.; 6 Division of Cardiology, Jill and Mark Fishman Center for Lymphatic Disorders, Children’s Hospital of Philadelphia, Philadelphia, Pennsylvania, USA.; 7 Department of Biomedical Engineering, Yale School of Medicine, New Haven, Connecticut, USA

**Keywords:** Immunology, Neuroscience, Lymph, Neuroimaging, Proteomics

## Abstract

The outflow of ‘dirty’ brain fluids from the glymphatic system drains via the meningeal lymphatic vessels to the lymph nodes in the neck, primarily the deep cervical lymph nodes (dcLN). However, it is unclear whether dcLN drainage is essential for normal cerebral homeostasis. Using dynamic contrast-enhanced magnetic resonance imaging (DCE-MRI) and computational fluid dynamics, we studied the impact of long-term mechanical stress from compromised dcLN drainage on brain solute and fluid outflow in anesthetized rats. We found that in young, but not middle-aged, rats, impairment of dcLN drainage was linked to moderately increased intracranial pressure and the emergence of extracranial perivenous drainage, with no evidence of hydrocephalus at any age. Surprisingly, both age groups showed enhanced brain solute clearance despite reduced glymphatic influx. CSF proteomic analysis revealed cellular stress in the form of low-grade inflammation and upregulation of pathways associated with neurodegeneration and blood brain barrier leakage in the rats with impaired lymphatic drainage. Our findings highlight that dcLN drainage is indeed a prerequisite for normal cerebral homeostasis in the rat and reveal the brain’s age-dependent compensatory responses to chronic impairment of its lymphatic drainage pathways.

## Abstract 2

The brain adapts to chronic lymphatic drainage impairment by accelerating solute clearance and diverting ‘dirty’ brain fluids to alternate egress routes.

## Introduction

The interconnected glymphatic and lymphatic systems are involved in brain waste clearance and CNS immune surveillance (1–6). In the glymphatic system, cerebrospinal fluid (CSF) enters from the brain’s periarterial spaces to the parenchyma, where it mixes with interstitial fluid (ISF) and picks up waste solutes (5, 7). This ‘dirty’ fluid exits the brain via perivenous routes and is collected by the meningeal lymphatic vessels (mLV) that drain via lymphatic collectors to the cervical lymph nodes (4, 6, 8–10). Combined, these interlinked processes are classified as glymphatic-lymphatic system “coupling” (7, 11). The functional connections between the glymphatic and lymphatic systems have been documented in both normal mice and Prox1-GFP (Prospero homeobox protein 1 green fluorescent protein) mice (6, 9). Although the glymphatic and lymphatic systems are interconnected, impairment of mLV drainage does not seem to adversely affect cerebral fluid homeostasis. For example, the absence of mLV in transgenic mice with defective vascular endothelial growth factor (VEGF)-C/D-VEGF receptor-3 signaling (6), or ablation of the dorsal cranial mLV via photodynamic therapy with light-activated verteporfin (8) were not associated with increases in the intracranial pressure (ICP). When it comes to the association between mLV drainage and neurodegeneration, the evidence is mixed. There is substantial support for both sides of the argument, making this area of research currently inconclusive (12–14).

We have been interested in understanding how different mechanical stressors such as hydrocephalus (15) or augmentation of respiratory function (16) impact glymphatic-lymphatic coupling. For these studies, we developed dynamic contrast enhanced magnetic resonance imaging (DCE-MRI) and computational fluid dynamics modeling to capture influx and clearance via the glymphatic perivascular system as well as drainage to the cervical lymph nodes (16, 17). Here, we applied these tools to shed more light on the processes the brain might engage in in the setting of chronic mechanical stress caused by compromised drainage to the deep cervical lymph nodes (dcLN) in the rat. In addition, we performed quantitative CSF proteomics to explore how this mechanical stress may affect the brain’s proteome, as reflected by the CSF, biochemically.

We found that in 3-month-old (3 M) rats, chronic dcLN drainage impairment was associated with dysregulation of cerebral fluid homeostasis manifesting as moderately increased ICP and aberrant drainage along extracranial perivenous pathways. In middle-aged (10 M) rats, dcLN drainage impairment was associated with increased drainage along the external carotid arteries and ICP was normal. Furthermore, the DCE-MRI brain data revealed that in rats with impaired drainage, glymphatic influx was reduced while, surprisingly, clearance was accelerated. CSF proteomic analysis from the young and middle-aged rats with impaired dcLN drainage indicated a signature of mild inflammation, as well as upregulation of pathways associated with cellular stress, blood brain barrier (BBB) leakage, and neurodegeneration. Collectively, our results show that, in young rats, the brain reacts to mechanical stress from impaired dcLN drainage by both engaging alternate drainage pathways and accelerating solute clearance. In middle-aged rats, this adaptive response is significantly blunted compared with younger rats. However, despite these compensatory changes, the dcLN intervention still resulted in low-grade inflammation and a general signature of impaired cellular proteostasis, implying that long-lasting deterioration in lymphatic drainage is conducive to cellular damage.

## Results

### dcLN drainage impairment in young and middle-aged rats.

**Figure 1:**
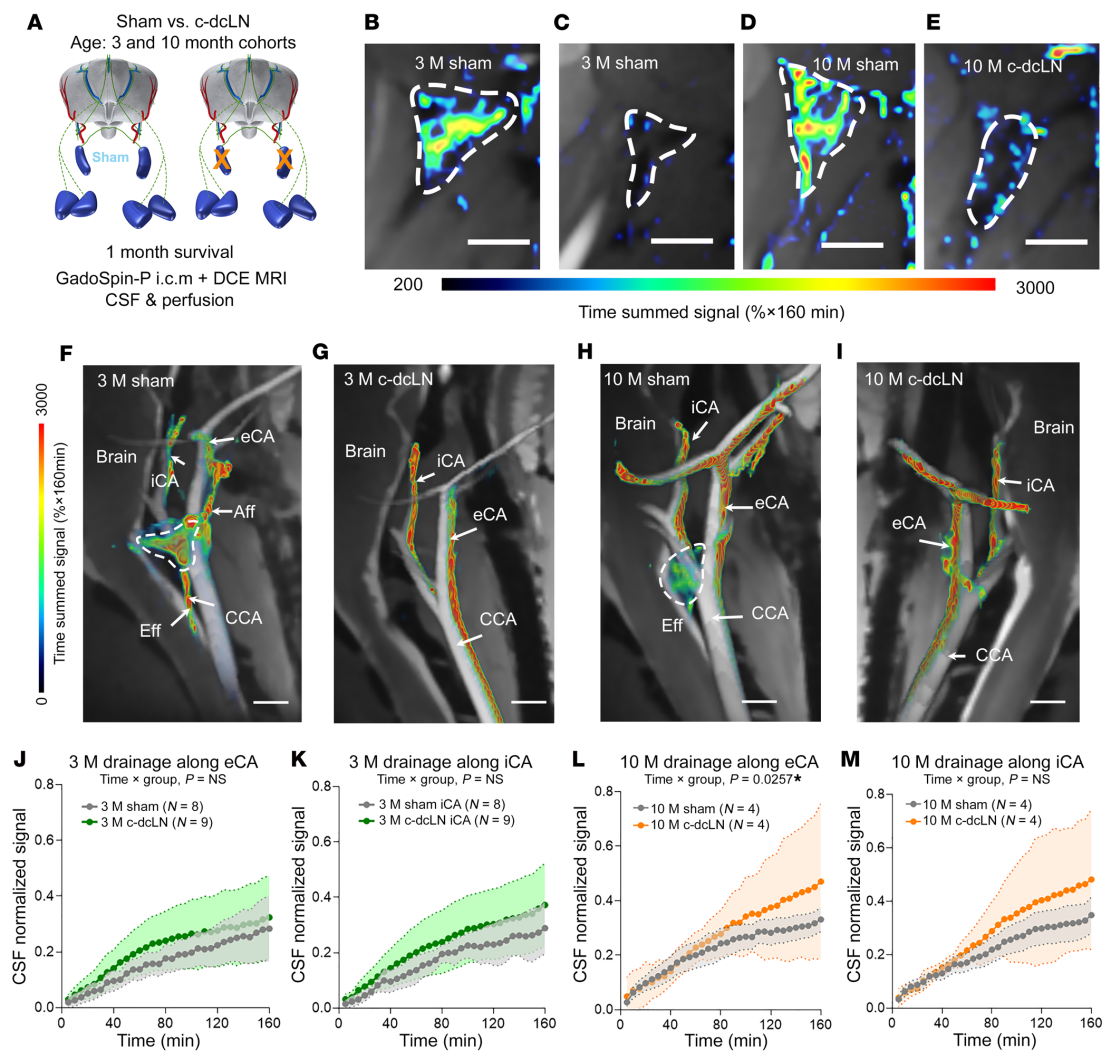
Evidence of dcLN drainage impairment in young and middle-aged rats. (**A**) Schematic overview of the experimental design. (**B** and **C**) Drainage to deep cervical lymph nodes (dcLN, dashed outlines) of 3 month old (3 M) rats after sham (**B**) or c-dcLN surgery (**C**). (**D** and **E**) Corresponding data from 10 M rats. Scale bars: 2 mm. (**F**) Drainage streams along the internal carotid artery (iCA) and external carotid artery (eCA). The dcLN is located immediately lateral to the common carotid artery (CCA). Aff, afferent; Eff, efferent lymphatic vessels. (**G**) Corresponding data from a 3 M c-dcLN rat in which drainage along the iCA and eCA is sustained, although the dcLN is gone. (**H** and **I**) Corresponding sham and c-dcLN data from 10 M rats. Scale bars: 2 mm. (**J** and **K**) Time signal curves (TSC) of drainage along the eCA (**J**) and iCA (**K**) from 3 M cohorts. Corresponding graphs of drainage along the eCA and iCA from the 10M cohorts. Data are presented as mean and 95% CIs. A linear mixed model for repeated measures with (FDR) correction for multiple comparisons was utilized to assess differences across groups. *P* values for time × group interaction effects are indicated on the graphs. Note that drainage along the eCA of 10 M c-dcLN rats is increased compared with sham rats. *P < 0.05. Corresponding graphs of drainage along the eCA and iCA from the 10M cohorts (**L** and **M**).

To study the long-term effects of lymphatic drainage impairment on fluid flows in the brain, we utilized a rat model of electrical cauterization of the dcLN, with sham surgery consisting of only node exposure. Cohorts of young adult (3 M) as well as middle-aged (10 M) rats were included to explore age-related differences in GadoSpin-P drainage from the CSF/brain to the cervical lymph nodes (Figure 1A). One month after dcLN or sham surgery, DCE-MRI images at the level of the neck were acquired while the rats were under anesthesia with dexmedetomidine and low-dose isoflurane, (16, 17). In both 3 M and 10 M sham rats, the dcLN appeared structurally intact and exhibited high signal intensity, most often extending into the efferent lymphatic vessels, suggesting that the nodes both received and drained GadoSpin-P (Figure 1, B and D). In contrast, in the c-dcLN rats of both age groups, the dcLN were dark and shrunken with no substantial signal enhancement (Figure 1, C and E). To quantify cervical lymph node drainage, we extracted the time signal curves (TSC) from the dcLN, accessory cervical lymph nodes (Acc LN), and submandibular cervical lymph nodes (SMLN). The TSC revealed that the cauterization procedure reduced dcLN drainage by approximately 80% in both 3 M and 10 M cautherized-dcLN (c-dcLN) rats (Supplemental Figure 1, A, D, and G; supplemental material available online with this article; https://doi.org/10.1172/JCI182555DS1). The TSC analysis revealed no differences in drainage to either the Acc LN or the SMLN across groups (Supplemental Figure 1, B, C, E and F).

We previously documented that GadoSpin-P drains from the CSF/brain towards the dcLN via ‘streams’ running along the external carotid artery (eCA) (17). In sham rats, we observed that the afferents to the dcLN appeared to originate from the eCA streams (Figure 1, F and H). Animal studies have shown that interstitial fluid can also flow in the adventitia associated with large arteries, veins, and peripheral nerves (18). It is plausible, therefore, that the streams observed along the neck vasculature represent such adventitial flow. In the c-dcLN rats, drainage streams along the eCA as well as the iCA continued to flow in spite of no visible dcLN (Figure 1, G and I). To assess the stream ‘strength’ across groups, we normalized the DCE-MRI data to the peak CSF signal. The TSC of the eCA and iCA streams appeared as monotonically increasing signal curves in both age groups (Figure 1, F–I). In the 3 M rats, the mean signal along the eCA and iCA increased to approximately 30%–40% of the peak CSF signal at 160 minutes with no differences across sham and c-dcLN rats (Figure 1, J and K). In the middle-aged cohorts, the TSC along the eCA of the c-dcLN rats was significantly increased compared with sham rats (*P* = 0.0257, Figure 1L). The TSC along the iCA of the 10 M rats trended to be higher in the c-dcLN group (*P* = 0.1025, Figure 1M). From these data, we concluded that dcLN cauterization significantly impaired dcLN drainage in both age groups. Furthermore, drainage along the eCA and iCA continued to flow in the c-dcLN rats, and drainage along the eCA was significantly increased in the 10M c-dcLN rats.

### dcLN drainage impairment is associated with aberrant extracranial drainage and increased intracranial pressure in young rats.

**Figure 2:**
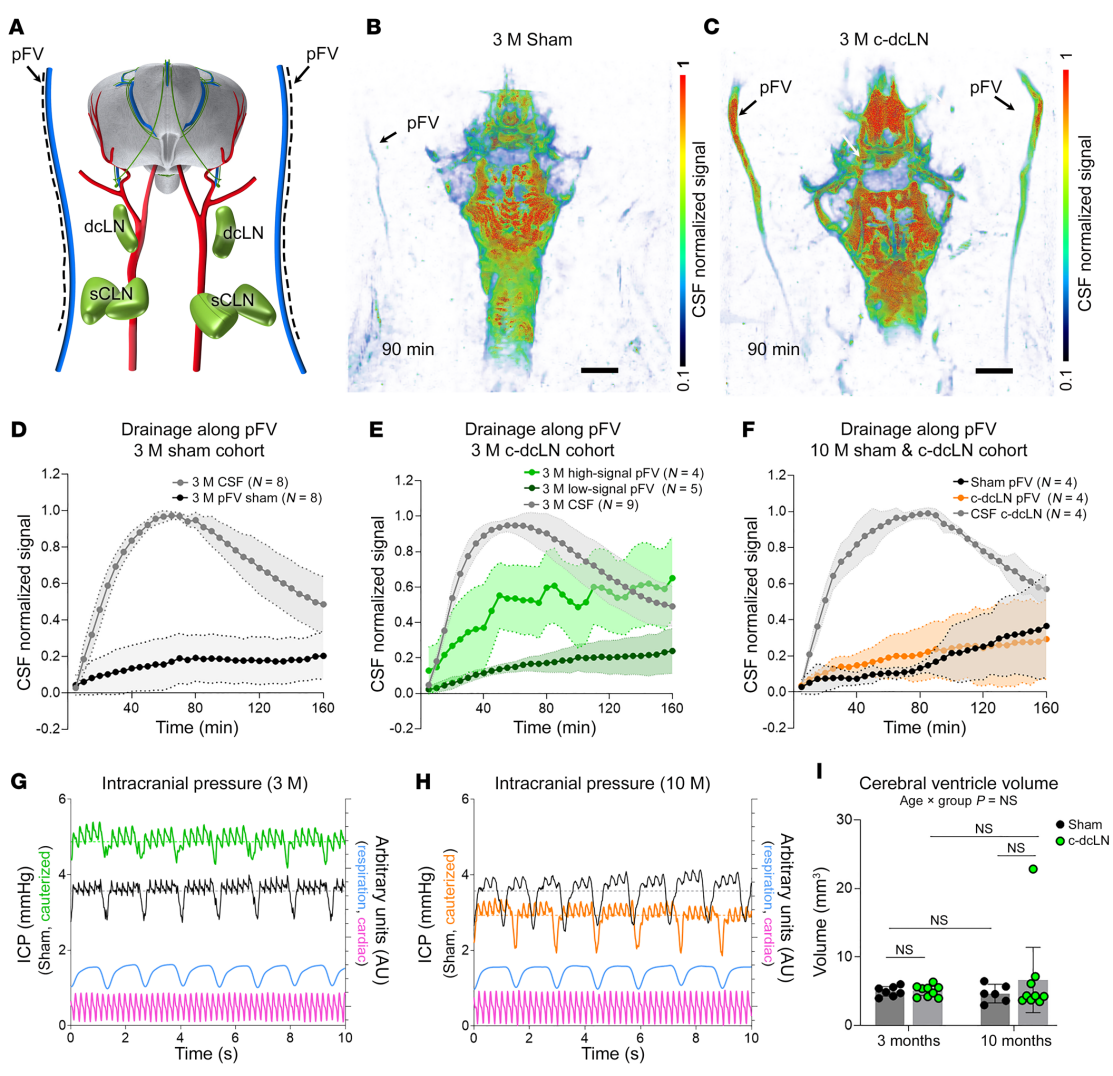
Chronic dcLN drainage impairment perturbs cerebral fluid homeostasis. (**A**) Illustration of the rat brain in relation to carotid arteries (red), deep cervical lymph nodes (dcLN), superficial cervical lymph nodes (sCLN), and posterior facial veins (pFV). The black dashed lines illustrate streaming along the pFV. (**B**) 3D maximum intensity projection (MIP) image from a 3-month (3 M) sham rat. (**C**) Corresponding 3D MIP image from a 3 M c-dcLN rat. Scale bar: 3 mm. (**D**) Time signal curves (TSC) of drainage along the pFV of 3 M sham rats. (**E**) Corresponding TSC data from the 3 M c-dcLN rats. TSC from c-dcLN rats with low-signal (dark green) and high-signal (bright green) streams. A significant time × group interaction effect across the rats with low versus high pFV streams in 3 M c-dcLN rats (*P* = 0.001). (**F**) Corresponding data from the 10 M cohorts. Data are presented as mean and 95% CIs. (**G**) Representative intracranial pressure (ICP) waveforms from a 3 M sham rat (blue) and a 3 M c-dcLN rat (orange) with moderately higher ICP. (**H**) Corresponding data from the 10 M cohort showing similar range ICPs in sham and c-dcLN rats. (**I**) Graphs of the cerebral ventricular volume from the 3 M and 10 M cohorts. Each dot represents data from one rat. Data are presented as mean and 95% CIs. Statistical analysis used a linear mixed model for repeated measures with FDR correction for multiple comparisons.

The DCE-MRI images acquired at the neck level capture the vasculature of both the head and neck, including the posterior facial vein (pFV), which drains the temporal sinuses and scalp (19). In the young, 3 M, cohort, we noted streams with strikingly strong signal along the pFV in 4 of the 9 c-dcLN rats, while none were observed in the sham rats (Figure 2, A–C, G, H). The signals of the pFV streams in the 3 M sham rats were of low magnitude and increased slowly to approximately 20% of peak CSF signal levels, while those of the aforementioned 4 c-dcLN rats increased rapidly to approximately 60%–70% of peak CSF signal levels (the other 5 young c-dcLN rats exhibited low signal pFV streams) (Figure 2, D and E). Analysis revealed a significant time and group interaction effect across the rats with low versus high pFV streams in the 3 M c-dcLN rats (*P* < 0.001). In the 10 M rats, no differences in signal intensity of the pFV drainage streams were observed across the sham and c-dcLN groups (*P* = 0.258, Figure 2F).

To further explore the physiological effects of chronic dcLN drainage impairment, we measured ICP at the level of the cisterna magna. We observed a moderate ICP increase in the young c-dcLN (3 M sham (*n* = 8), ICP: 3.79 ± 0.94 mmHg versus 3 M c-dcLN (*n* = 7), ICP: 8.81 ± 3.70 mmHg, mean difference = 5.02 mmHg, 95% CI = [1.60, 8.44] mmHg, *P* = 0.017) but not in the middle-aged rats (10 M sham (*n* = 3) ICP: 5.84 ± 4.34 mmHg versus 10 M c-dcLN (*n* = 5) ICP: 3.83 ± 2.56 mmHg, mean difference = –2.00 mmHg, 95% CI = [–11.02, 7.01] mmHg, *P* = 0.521). We also examined the anatomical proton density weighted MRI scans of the cohorts to screen for potential hydrocephalus. None of the 3 M rats exhibited changes in ventricle volume (Figure 2I). One 10 M rat in the c-dcLN group exhibited severe hydrocephalus in addition to a small cerebral microbleed; however, the cerebral ventricle volume in the remaining rats were within normal limits (Figure 2I). Overall, our rat model shows that dcLN drainage impairment is not associated with hydrocephalus, irrespective of age. Additionally, moderately increased ICP and frequent aberrant extracranial drainage are observed in young c-dcLN rats, but these conditions are not present in middle-aged rats.

### Accelerated glymphatic solute clearance in rats with compromised dcLN drainage.

We next characterized the impact of the c-dcLN procedure on the brain’s glymphatic transport. To measure brain-wide glymphatic influx and clearance, we acquired DCE-MRI images with CSF administration of gadoteric acid (Gd-DOTA, MW 539 Da) (17, 20) in separate series of 3 M and 10 M cohorts. The unbalanced regularized optimal mass transport (urOMT) framework used to analyze the DCE-MRI brain images allowed us to distinguish between influx (tracer transport from the CSF into brain parenchyma) and clearance (tracer exiting from the brain) (21). Supplemental Figure 2 presents an overview of the processing pipeline. Briefly, the urOMT analysis creates a time series of 3D whole-brain maps, representing a metric known as “*r-flux*,” from each rat’s DCE-MRI data set, where voxels are color coded: red indicates influx rates and blue indicates clearance rates (Supplemental Figure 2B). Over time, the red colors become lighter, indicating declining influx rates, and eventually turn blue as clearance in the tissue becomes predominant. (Supplemental Figure 2B). From the *r-flux* maps, changes in volume fractions of influx/clearance in a given ROI can be obtained at each time point (Supplemental Figure 1, C and D). The graph displays the time-dependent changes in the brainstem volume fractions exhibiting influx and clearance (Supplemental Figure 2D). The other metric derived is the regional *net* rates calculated as the spatial average of all *r-flux* rates in the ROI. As an example, we show the time trajectory of the net rate in the brainstem (Supplemental Figure 2E) demonstrating high, positive net rates over the first 120 minutes (influx dominates) followed by a decrease (clearance dominates). In the brainstem of this particular rat, the mean net rate from 30 → 110 minutes is approximately 0.43 % per minute and from 120 → 160 minutes it drops to approximately 0.04% per minute (Supplemental Figure 1E). This approach was applied to analyze influx and clearance kinetics in the sham and c-dcLN rats.

**Figure 3:**
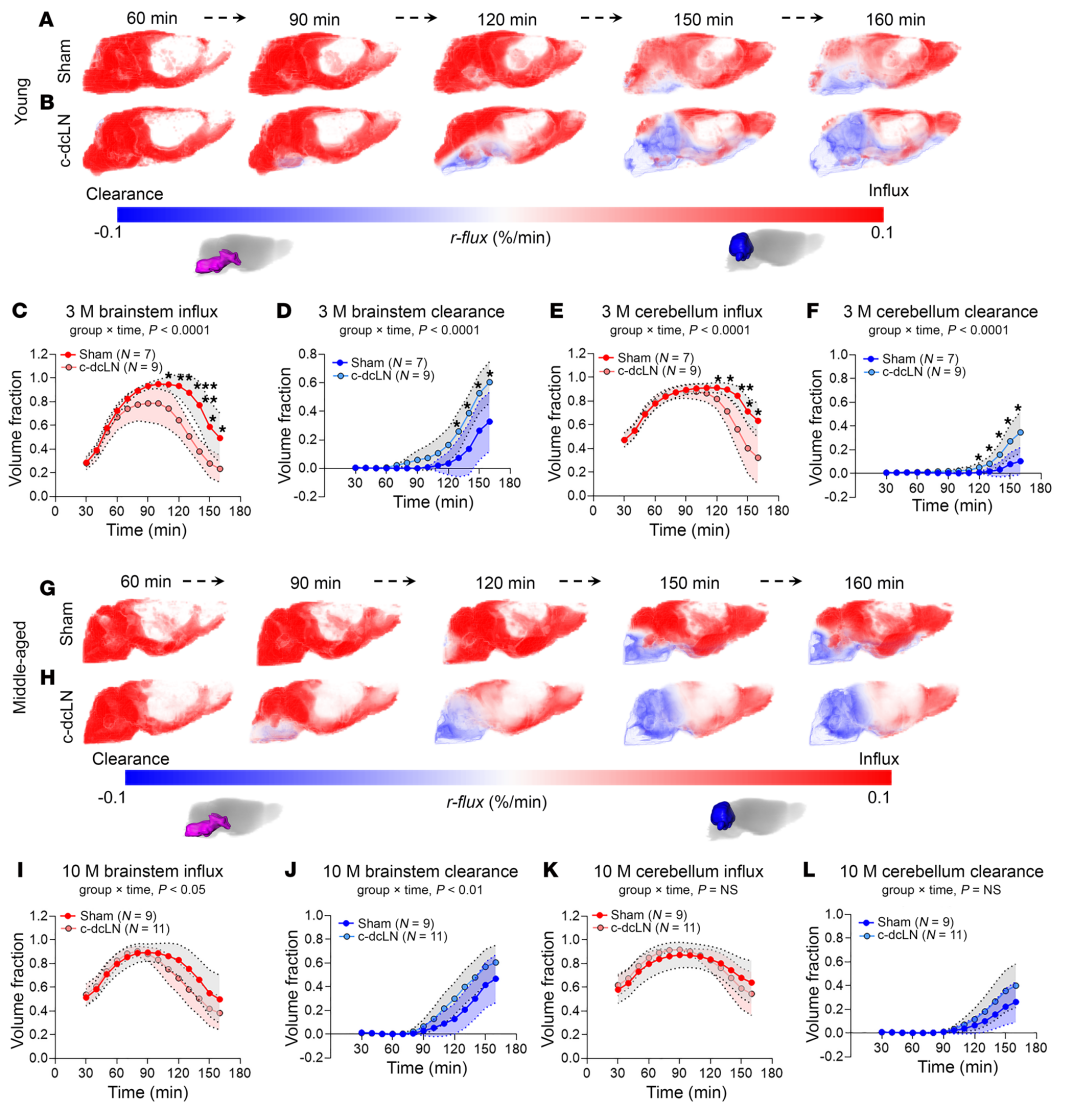
Accelerated solute clearance in rats with compromised dcLN drainage. (**A** and **B**) Time series of representative *r-flux* maps from a 3 M sham rat with intact dcLN drainage (**A**) and a 3 M c-dcLN rat with impaired drainage (**B**). Positive *r-flux* values in red denote influx and negative *r-flux* values in blue indicate clearance. Note that tissue clearance in the c-dcLN rats manifests earlier (~120 min) compared with sham rats (~150 min). Scale bar: 3 mm. (**C**–**F**) Graphs showing the time trajectories of influx and clearance as fractional volumes from the brainstem and cerebellum across the 3 M sham (*n* = 7) and c-dcLN (*n* = 9) rats. The regional volume fractions of influx and clearance are shown in red and blue, respectively. (**G** and **H**) Corresponding representative *r-flux* maps from 10 M cohorts. (**I**–**L**) Corresponding graphs showing the time trajectories of influx and clearance as fractional volumes from the brainstem and cerebellum across the 10 M sham (*n* = 9) and c-dcLN (*n* = 11) rats. Data are presented as mean and 95% CIs. A linear mixed model for repeated measures with FDR correction for multiple comparisons was utilized to assess differences across groups. For each region, *P* values for time × group interaction effects are indicated below the graphs; *P < 0.05, **P < 0.01, ***P < 0.001.

The time series of *r-flux* maps revealed distinct patterns between the sham and c-dcLN groups. In a representative 3M sham rat, influx dominated for approximately 120 minutes, after which clearance became evident, particularly in regions near the skull base (Figure 3A). Conversely, in a 3 M c-dcLN rat, the time-dependent *r-flux* distribution pattern was markedly different, with an overall reduction in influx and earlier emergence of clearance (Figure 3B). The quantitative analysis of the *r-flux* maps from the 3 M cohorts focused on the brainstem and cerebellum and confirmed that influx was significantly reduced, and clearance emerged earlier (Figure 3, C–F). For the 10 M cohorts, the visual distribution pattern of influx and clearance on the *r-flux* maps appeared similar to the 3 M groups (Figure 3, G and H). In the 10 M cohort, the time trajectories of the influx and clearance volume fractions from the brainstem were also significantly different across groups, with clearance emerging earlier in the c-dcLN rats (Figure 3, I and J). There were no differences in either cerebellar influx or clearance across the 10 M groups (Figure 3, K and L).

We also examined the corresponding mean net rates during the influx- and clearance-dominated phases across the groups. In the brainstem, the mean net rates during the influx phase were similar across ages and experimental groups (Supplemental Figure 3A). However, during the clearance phase, the mean net rate in the brainstem of the 3 M c-dcLN rats significantly decreased (*P* = 0.006) (Supplemental Figure 3B). In 10 M rats, there was a trend towards a decrease in the mean net rate of the brainstem during the clearance dominated phase (*P* = 0.072, Supplemental Figure 3B). In the cerebellum, both young and middle-aged rats exhibited similar mean net rates during the influx phase across sham and c-dcLN conditions (Supplemental Figure 3C). During the clearance phase, however, mean net rates significantly decreased in the young rats (Supplemental Figure 3D). These findings suggest that glymphatic system function in 3M c-dcLN rats is altered. Specifically, glymphatic influx is regionally reduced, while solute clearance commences earlier. In the middle-aged c-dcLN cohort, influx was reduced and clearance was accelerated in the brainstem only, indicating that these effects were not as widespread as in the young cohort.

### CSF proteomics suggest activation of low-grade inflammation and neurodegeneration.

**Figure 4:**
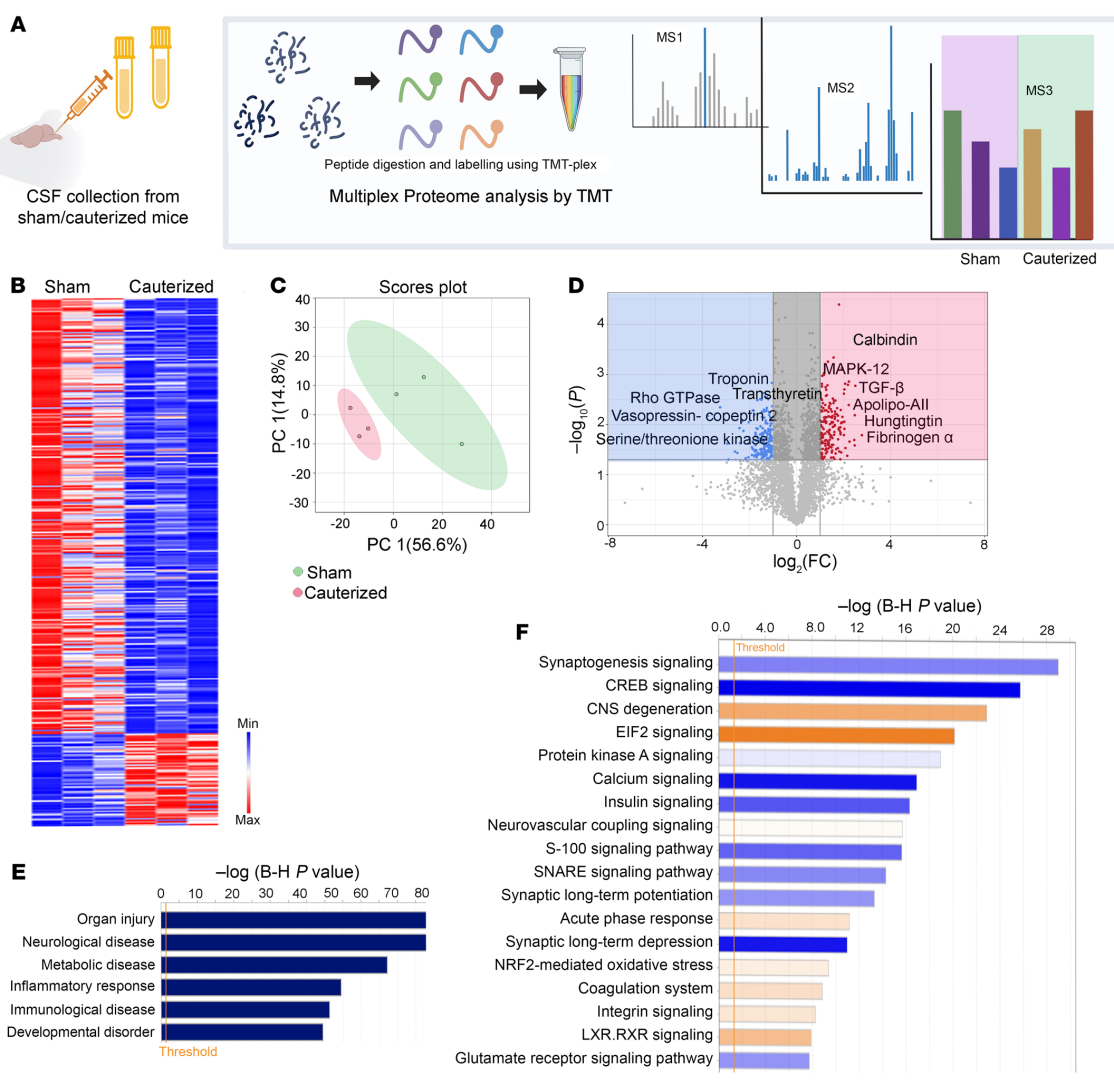
Inflammatory and degenerative signature in the CSF of young rats with compromised dcLN drainage. (**A**) Graphical abstract summarizing quantitative mass spectrometry–based proteomics with stable isotope–containing isobaric tags (MS3 notch method). (**B**) PEAKS analysis with Q-module generated hierarchically clustered heat map from c-dcLN (*n* = 3) and sham (*n* = 3) rats. The relative protein abundance in the 2 conditions is shown for the protein groups that passed the filters for protein identification (–log (*P* value) > 13 for protein ID, corresponding to *P* < 0.05 by ANOVA) and showed significant changes (*P* < 0.05 by ANOVA/*t* tests). The full list of proteins is reported in Supplemental Table 2. Positive values reflect fold increases (red color), and negative values reflect fold decreases (blue color). (**C**) Unsupervised 2D principal component analysis (2D-PCA) score plot generated from the analysis of CSF identified proteome listed in Supplemental Table 2 from sham (green) and c-dcLN (red), with principal components 1 (PC1) and 2 (PC2) accounting for 56.6% and 14.98% of the variance. (**D**) Volcano plot illustrating the significance of protein expression ratios in c-dcLN/sham. Statistically significant proteins (*P* < 0.05 by ANOVA) are highlighted in red for 2-fold up-regulated and in blue for 2-fold down-regulated proteins. These proteins are indexed in Supplemental Table 2. (**E**) IPA analysis–based enrichment of top pathways (*P* < 0.05 by Fisher’s exact test with Benjamini-Hochberg correction) observed in c-dcLN/sham. (**F**) Quantitative analysis of protein expression profiles and IPA assigned *z* score function to all eligible canonical biochemical and cellular pathways: where *z* < 2 (in blue shades) represents significant downregulation while *z* > 2.0 (in orange shades) represents a significant upregulation (Supplemental Table 2).

**Figure 5:**
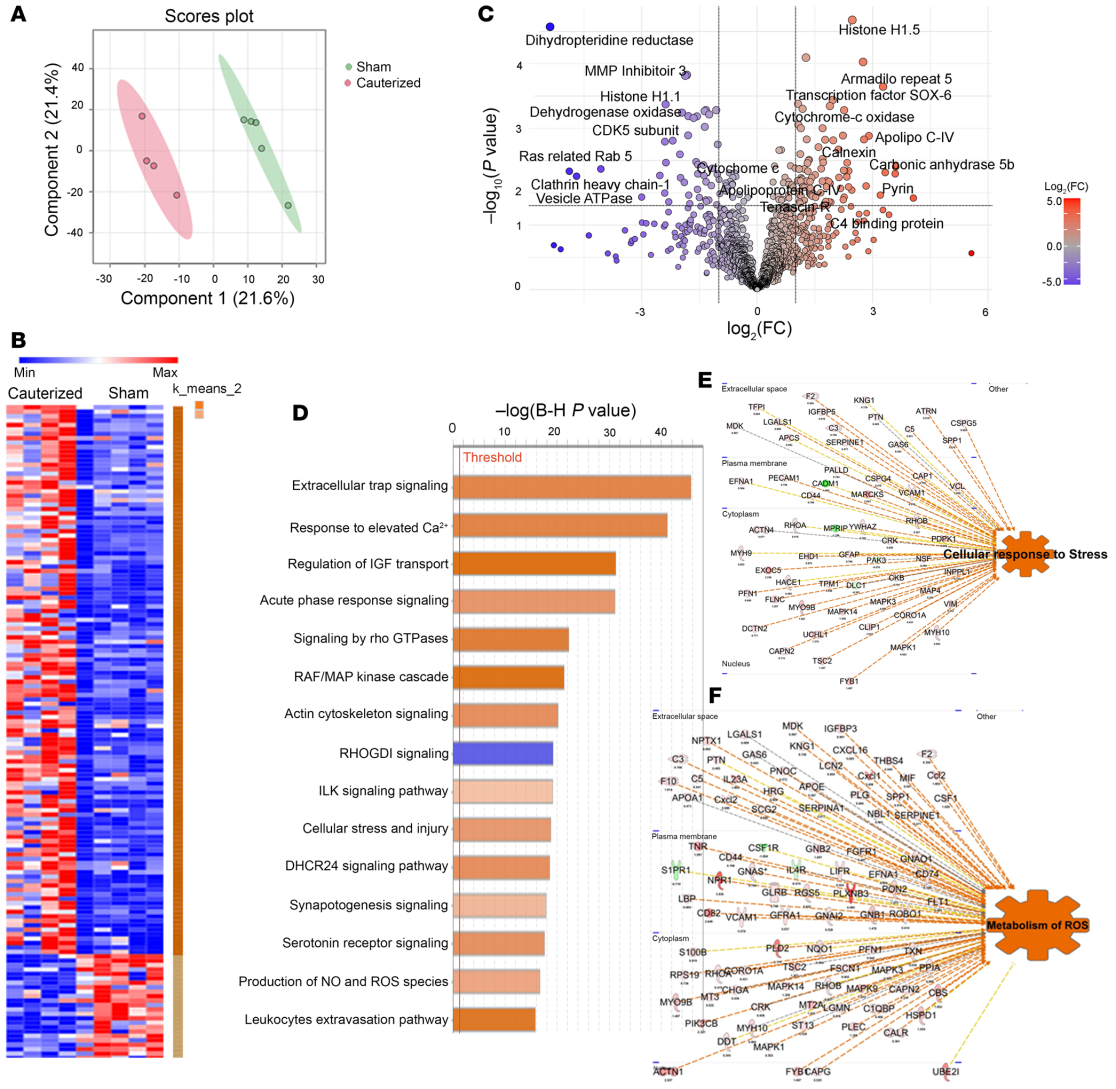
A proteomic signature of acute phase response and oxidative stress is present in the CSF of middle-aged dcLN-cauterized adult rats. (**A**) Partial least squares discriminant analysis (PLS-DA) displaying score plot based proteomic profile of CSF harvested from sham (green) and dcLN-cauterized (red) rats with ellipses representing the 95% CI of the group clustering in 10-month (10 M) adult cohort. (**B**) Heat map of representative biological replicates profile contrasting all FCs between the proteins identified in the sham and dcLN groups (Supplemental Table 2). Only proteins that passed a selected significance statistical threshold (ANOVA/*t* test applied in PEAKS, *P* < 0.05) are displayed in the heat map. (**C**) Volcano plot showing the significance in the ratio corresponding to protein expression in dcLN/sham; highlighted in red and green are the statistically significant proteins (*P* < 0.05 by ANOVA), showing 2-fold up- and downregulation, respectively (indexed in Supplemental Table 2). (**D**) Canonical pathway enrichment analysis using IPA (QIAGEN) assigned *z* score. The orange bar indicates a positive *z* score, which is an activated/upregulated pathway. Color depth represents the degree of the *z* score. The horizontal axis indicates −log (Benjamini-Hochberg *P* value), and the vertical axis represents the given pathways. Additional details are mentioned in Supplemental Table 2. (**E** and **F**) Quantitative IPA analysis; identified proteins are represented as gene symbols and are predicted to be upregulated (in red) or downregulated (in green) in the comparison between groups. For network generation, datasets containing gene symbols were uploaded into the IPA application along with their rescaled log_2_ transformation of average protein area ratios. Proteins with significant changes (*P* < 0.05, by ANOVA/ *t* tests) are reported in Supplemental Table 2.

**Figure 6:**
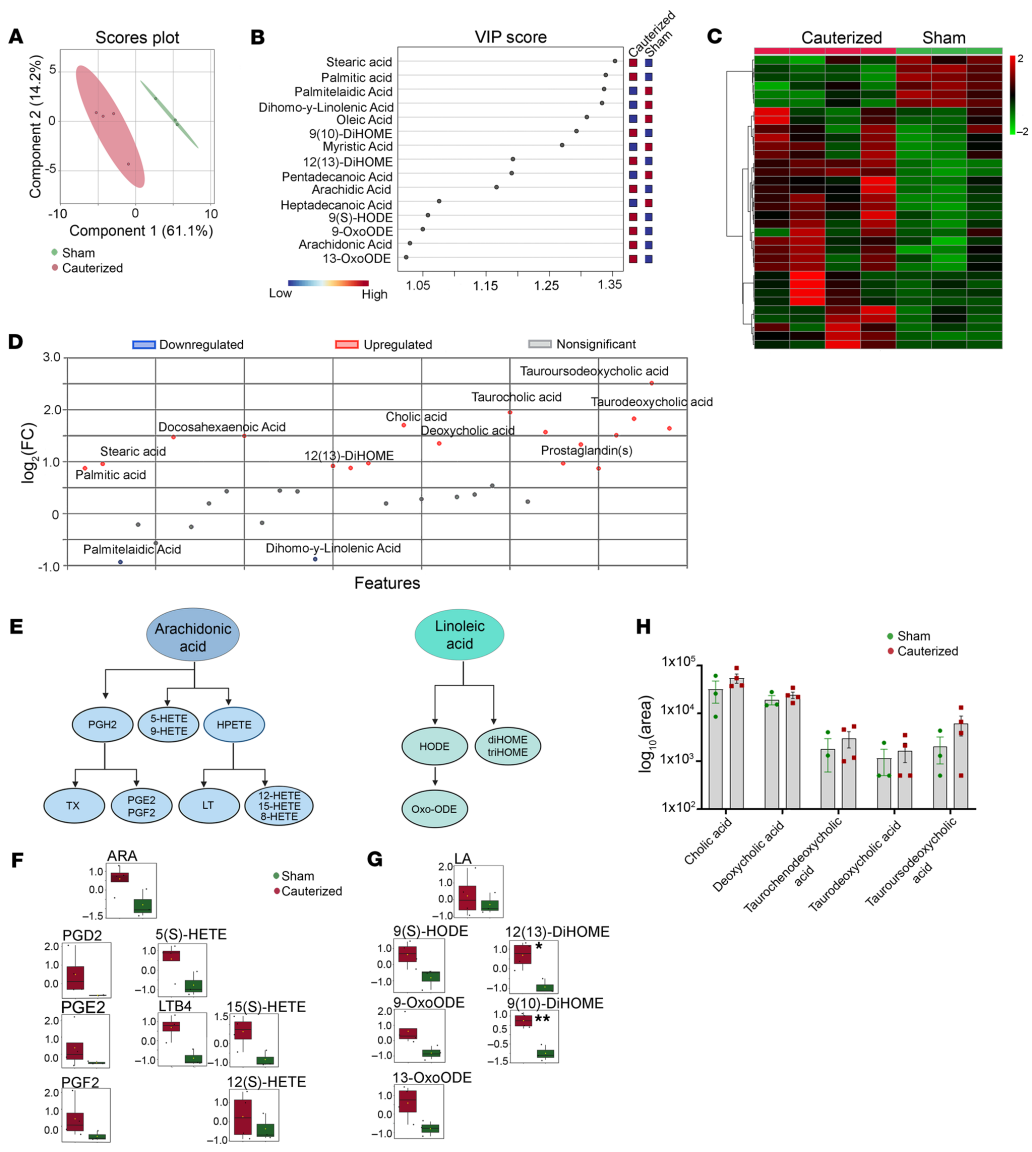
Oxylipins and bile acids are increased in the CSF of young rats with compromised dcLN drainage. (**A**) 2D PLS-DA score plot based on the lipidomic profile *(Oxylipins)* of CSF harvested from sham (green) and c-dcLN (red) rats highlighting the separation between the 2 groups with ellipses representing the 95% CIs of the group clustering. (**B**) Associated Variable Importance in Projection (VIP) scores ranking the most important metabolites (VIP > 1) that determine the separation in the PC1 dimension of the 2 sample groups. The colored boxes on the right side of the VIP score plot denote relative metabolite concentrations between the different groups. The red-blue gradient key indicates the gradient showing upregulation and downregulation. (**C**) Hierarchical cluster analysis (HCA) showing the deregulated metabolite profile in sham and dcLN groups. Each colored cell on the map corresponds to a normalized concentration value, with samples in columns and compounds in rows. Red and green colors indicate positive and negative correlations, respectively. (**D**) Deregulated pattern of quantified metabolites in the 2 groups where the statistical significance was calculated in MetaboAnalyst 6.0 using the built-in *t* test/ANOVA. (**E**) Overview of eicosanoids and oxylipin metabolism (**F** and **G**) levels of eicosanoid/oxylipin metabolites quantified in the CSF of dc-LN and sham rats. Boxplots depict relative concentration for the most significantly altered metabolites (*P* < 0.05). Y-axes are represented as relative units. (**H**) Relative abundance of bile acids mapped in the CSF of dc-LN and sham rats. Each point represents a biological sample. Statistical significance was determined using a 2-way ANOVA. All error bars represent SEM. *P < 0.05, **P < 0.01, ***P < 0.001 (Supplemental Table 3).

To gain a comprehensive understanding of how the mechanical stress from dcLN drainage impairment would impact the biochemical CSF profiles in the young 3 M as well as middle-aged 10 M rats, we harvested CSF for mass spectrometry analysis (LC-MS/MS) (Figures 4, 5, and 6, Supplemental Figures 4–7, and Supplemental Tables 2 and 3). Relying on an advanced in-depth proteomic approach; data-independent acquisition (DIA) and Tandem Mass Tags–based (TMT 10-plex–based) quantitative analysis, we captured sensitive biochemical changes in the CSF. In the young, 3 M, cohort (*n* = 6 CSF samples), TMT quantitative analysis identified a total of 4,089 proteins, of which 357 proteins were significantly up- and downregulated by *P* < 0.05) (Figure 4, A–D, and Supplemental Table 2A). PCA analysis revealed a clear separation between the groups, with the protein level variance of 71.4% in the first 2 principal components indicative of “omic” changes in the young rats with compromised dcLN drainage (Figure 4C). Among the significantly upregulated proteins were classical proteins associated with neuroinflammation, such as fibrinogen subunits (α, β, γ), complement subunits C5, C6, C8, and C9, apolipoproteins (A-I, A-II, B-100, C-I, C-II, D, M), integrins, damage-associated molecular patterns S-100b, heat shock protein 1, tissue remodeling related matrix metalloproteinases (MMPs), and proinflammatory prostaglandins (Figure 4, B and D, Supplemental Figure 4, and Supplemental Table 2, A and B). Proteins known to be derived from blood ultrafiltration including albumin, ceruloplasmin, and plasminogen were also upregulated, suggestive of barrier compromise (Supplemental Figure 4B). Additionally, candidate proteins involved in the initiation/progression of neurodegenerative cascades, such as calbindin, glial fibrillary acidic protein (GFAP), prion-derived proteins, TATA-box binding proteins, α/β-synucleins, VEGF, amyloid subunits, and tumor necrosis factors (TNFs) were also upregulated in the c-dcLN dataset (Supplemental Figure 4E and Supplemental Table 2). To get further insight into the underlying biological pathways, we employed IPA (ingenuity pathway analysis) enrichment (22). The analysis indicated potential associations among the detected CSF protein changes and pathways associated with disease-and-injury processes such as organ damage, CNS-disease, and metabolomic disorders (Figure 4E and Supplemental Figures 4 and 5). Unbiased analysis also revealed significantly upregulated cellular processes and/or pathways related to degeneration, stress-related eukaryotic initiation factor 2 (EIF2) signaling, acute phase response, LXR/RXR signaling (lipid homeostasis), and activation of NRF2-mediated signaling (Figure 4F), pointing to stress-induced exacerbated inflammation. Among the pathways downregulated were synaptogenesis signaling, CREB signaling, calcium signaling, SNARE, and glutamate receptor signaling—key regulators of neurotransmission—as well as insulin receptor signaling and synaptic depression/potentiation, which are associated with broader brain function. These changes imply impaired neurotransmission and compromised brain activity (Figure 4F and Supplemental Figure 4F). IPA clusters the proteins identified as being differentially expressed, allowing a prediction of the resultant activation/inhibition status of entire networks, canonical pathways, and biological processes. Notably, the analysis predicted pathways related to neuronal activity, vasculogenesis/angiogenesis, and overall brain homeostasis as being significantly downregulated, indicating cellular distress in c-dcLN rats (Figure 4F and Supplemental Figures 4 and 5). Altogether, the CSF quantitative proteomic analysis highlights an early, low-grade inflammatory and neurodegenerative-like signature in the context of impaired brain dcLN drainage in young adult animals.

In the middle-aged, 10 M, cohort, DIA mass spectrometry identified 1,386 proteins, with 153 significantly deregulated (Supplemental Table 2F). Partial least-square discriminant analysis (PLS-DA) determined a PC1 and PC2 of 21.6% and 21.4%, respectively (Figure 5A and Supplemental Table 2K). Differentially expressed proteins, visualized using a volcano plot and a heat map, revealed significant differences between the 2 groups (Figure 5, B and C; Supplemental Table 2K and L). Among the significantly upregulated proteins, several key candidates stood out, notably, Histone H1.5, a variant of the linker histone H1 family, previously characterized as neurotoxic in chronic neurodegenerative diseases (23), BRCA1, known for its essential role in DNA repair, and cellular antioxidant defense (24, 25), and Pyrin, a component of the NLRP3 inflammasome complex. Additionally, mitochondrial enzymes such as COX6C, SOX-6, and COQ7, which are essential for mitochondrial electron transport and ATP production, exhibit deregulated profiles linked to mitochondrial and cellular stress (26). Armadillo repeat-containing protein 5 (ARMC5), which plays crucial roles in cell adhesion, signal transduction, and regulation of cellular processes, is also released into the CSF because of glial cell injury (27). Furthermore, matrix metalloproteinase-3 (MMP-3), a key mediator in brain injury and an indicator of BBB disruption, along with several subunits of the neuroinflammatory cascade proteins released after injury, were significantly altered in the middle-aged c-dcLN group (Figure 5, B and C) (28–30). Other notable proteins included Calnexin and Calpain, which play key roles in neuropathological events following traumatic brain injury and serine/threonine kinases, crucial in neuronal apoptosis and injury responses (Figure 5, B and C). Sortilin-1, known to rise significantly following brain injuries such as subarachnoid hemorrhage and Visinin-like protein 1, a neuronal calcium-sensor protein and established marker of neuronal injury, were also significantly upregulated (Figure 5, C–F, and Supplemental Table 2). To further understand the biological and molecular interactions in the identified proteins, an IPA-based enrichment/network analysis was performed (Figure 5, D–F, and Supplemental Figure 6C). Among the top significantly upregulated pathways were presence of extracellular traps, interleukin signaling, oxidative stress, leukocyte extravasation, and acute phase responses (Figure 5, D–F). The existence of an inflammatory milieu was further confirmed by the CSF presence of damage-associated molecular patterns (DAMPs), inflammatory biomarkers, chemokines, enzymes and proteases, acute phase response proteins, as well as astrocytic and neuroinflammatory markers quantitatively more abundant in the young as compared to the 10 M middle-aged rats (Supplemental Figure 6, A, B, and F).

### Evaluation of potential protein biomarkers for impaired lymphatic drainage.

To analyze CSF potential biomarkers of impaired lymphatic drainage, a multivariate analysis and PLS-DA was conducted in MetaboAnalyst Software 6.0 (Supplemental Figure 6, D and E, and Supplemental Table 2M). Based on the differently expressed protein, a multivariate exploratory ROC analysis was conducted utilizing the PLS-DA as a classifier and feature ranker (Supplemental Figure 6E). As evident, the highest AUC value of 0.97 (with 95% discriminatory performance) was obtained for the 6th biomarker model, having the highest predictive accuracy of 74% (Supplemental Figure 6D and Supplemental Table 2M). The frequency plot of the top 15 significantly dysregulated proteins identified protein biomarkers distinguishing the c-dcLN from sham rats. Transcription factor SOX-6, Dihydropteridine reductase, Histone H1.5, and Cytochrome c oxidase subunit 6C-2 are the top 4 potential biomarkers with the highest frequency rank and importance in the analysis (Supplemental Figure 6E). All these markers were previously associated with neurodevelopmental disorders (31) as well as neurodegeneration (32, 33).

### Metabolomics CSF analyses complemented the inflammatory phenotype in young rats.

The CSF metabolome equally offers an amenable platform to interpret brain function following an injury/damage (Figure 6, Supplemental Figure 7 and Supplemental Table 3). Multivariate analysis was conducted based on the metabolite concentration data, which showed overall group differences in the young rats (Figure 6 and Supplemental Figure 7). Using PLS-DA analysis (VIP > 1.0), we found 20 metabolites with a statistically different expression between the 3 M c-dcLN and sham groups (Figure 6, A and B, and Supplemental Figure 7). Among those, an accumulation of arachidonic acid (AA) was revealed (Figure 6, E and F), which, together with its downstream metabolites, has been reported to be increased in neuroinflammation, excitotoxicity, and Alzheimer’s disease (AD) (34, 35). Accumulation of short chain acyl carnitines C4 and C5, prominent intermediates of fatty acid metabolism, and several γ-glutamyl species, associated with glutathione de novo synthesis, was also observed in the c-dcLN rats. (Supplemental Figure 7, D and E) (36–38). On the other hand, sphingosine and sphingosine-1-phosphate, involved in CNS growth and differentiation, were downregulated following dcLN cauterization (Supplemental Figure 7D). Interestingly, biliverdin and its generating enzyme, biliverdin reductase, known to be upregulated in AD were also more represented in the CSF of c-dcLN rats (Supplemental Figure 7F). Finally, a signature of BBB leakage was also mapped in the young c-dcLN group (Supplemental Figure 7G).

### Lipidomics analyses reveal an increase in inflammatory oxylipins species in CSF.

Neuronal lipids are major structural components of the cell membrane and have a pivotal role in neuronal transmission. To further analyze the dcLN cauterization–induced homeostatic imbalance at the metabolomics level, we performed a lipidomics analysis to characterize the 3 M CSF-profile of bioactive lipids (Figure 6, Supplemental Figure 7, H and I, and Supplemental Table 3). Highly enriched polyunsaturated fatty acids (PUFAs) are abundant in the brain, and, upon redox imbalance and oxidation, they are metabolized to oxylipins, via enzymatic and nonenzymatic routes (39). CSF collected from young, 3 M rats (*n* = 4 c-dcLN, *n* = 3 sham) was subjected to targeted metabolomics to evaluate the oxylipins levels between the 2 experimental groups (Figure 6). A supervised PLS-DA revealed separation of the study groups while the generated Variable Importance in the Projection (VIP) plots identified the significant metabolites responsible for group separation (Figure 6, A–C). Several important metabolite candidates found to be significantly deregulated included stearic acid (*P* = 0.0001) and palmitic acids (*P* = 0.0004) in the c-dcLN rats (Figure 6D). Additionally, a targeted analysis revealed an increase in levels of a few of the proinflammatory oxylipins, including eicosanoids and linoleic acid oxidized derivative prostaglandins, and diHOMEs in the c-dcLN samples (Figure 6, E–G). The increase in AA was accompanied by an increase in leukotrienes, prostaglandin D2, E2, F2, and hydroxyeicosatrienate (HETE) derivatives (5(S)/15(S)/12(S)-HETE) (Figure 6, E and F). Furthermore, a decrease in linoleic acid was accompanied by a significant increase in dihydroxyoctadecenate (12(13) diHOME, *P* = 0.013, 9(10) diHOME, 0.002) along with an increase in hydroxyoctadecadienate (9(S)-HODE) and oxooctadecadienates (9-oxoODE, 13-oxoODE) (Figure 5G), without having visible differences in the concentrations of free fatty acid species (saturated, unsaturated) and glycophospholipids. (Supplemental Figure 7, H–J, and Supplemental Table 3). Notably, an accumulation of bile acids was also observed in c-dcLN compared with sham rats (Figure 6H).

## Discussion

In this study, we demonstrated that chronic dcLN drainage impairment leads to adaptations related to cerebral fluid homeostasis in both young and middle-aged rats. These changes are evidenced by the emergence of extracranial drainage pathways, regional reductions in glymphatic influx, along with accelerated clearance and increased ICP in the younger c-dcLN rats. We speculate that the extracranial drainage in the younger rats with impaired dcLN drainage was driven by moderately increased ICP. Importantly, there was no evidence of hydrocephalus in rats with chronic dcLN drainage impairment. We propose that, in rats with dcLN drainage impairment, the extracranial fluid flow, such as increased drainage along the pFV and/or carotid arteries, likely served to prevent major ICP increases. The moderate ICP increase observed in the young c-dcLN rat model contrasts previously published observations of normal ICP under conditions of impaired or absent mLV (6, 40). For example, in mice with genetic modifications that functionally impair mLV, the ICP was reported to be within normal range (6, 9). However, these previous studies were performed in mice, which, having a thinner skull, may prove more resilient to mechanical stress than the rat.

We documented that the response to chronic dcLN drainage impairment was more pronounced in young than in middle-aged rats. In young c-dcLN rats, influx was significantly reduced, and solute clearance was more accelerated in the brainstem and cerebellum when compared to the middle-aged c-dcLN cohort. Typically, a reduction in solute influx, as measured by in vivo DCE-MRI (41–45) or tracer uptake using ex vivo techniques (5, 46–48), is interpreted as an impairment of glymphatic function. However, the urOMT analysis allowed us to examine both influx and clearance, and, using *r-flux* maps, revealed that the net clearance rate was overall more accelerated in young than in middle-aged c-dcLN rats. The urOMT results also indicated a more widespread and accelerated clearance in the younger c-dcLN rats, which might reflect BBB compromise. In this scenario, the tracer is cleared via blood. Supporting this, the proteomic analysis of the CSF showed signs of BBB leakage in both young and middle-aged groups.

The omics data suggest that chronic dcLN drainage impairment led to an early, low-grade inflammatory and neurodegenerative-like signature in young rats, with less pronounced effects in middle-aged rats. Younger animals exhibited a more robust, proinflammatory, and neurodegenerative-like proteomic profile, possibly due to a more reactive neuroimmune environment. In contrast, older animals showed selective upregulation of stress and injury-related proteins, suggesting less widespread compensatory effects. This may reflect age-associated alterations in immune surveillance, drainage capacity, or repair mechanisms. Additionally, in an exploratory analysis, we also identified potential biomarkers in 10 M rats, demonstrating high clinical relevance, as evidenced by an AUC value of 0.97. This age group is particularly pertinent due to the documented decline in lymphatic-glymphatic function associated with aging, making the middle-aged rats a closer model to human patients suffering from diseases characterized by impaired brain-to-lymph drainage. Among the top 4 biomarker candidates were Sox transcription factors (TFs), which regulate critical processes in adult tissues, such as cell survival, regeneration, apoptosis, and homeostasis. Dihydropteridine reductase (DHPR) plays an essential role in tetrahydrobiopterin (BH4) metabolism, a cofactor necessary for synthesizing neurotransmitters like dopamine, serotonin, and norepinephrine. Deficiency or dysfunction of DHPR can lead to significant neurological issues and has been studied as a potential biomarker for brain damage (49). Histone H1.5, a linker histone variant, contributes to brain damage pathophysiology through its extracellular release from injured neural tissue, inducing neurotoxic and proinflammatory effects (23). Cytochrome c oxidase subunit 6C-2 (COX6C) is a key component of mitochondrial oxidative phosphorylation, central to cellular energy metabolism. These biomarkers represent critical molecular pathways involved in brain injury and neurological diseases, including transcriptional regulation (Sox TFs), neurotransmitter synthesis (DHPR), chromatin-related neurotoxicity and inflammation (Histone H1.5), and mitochondrial bioenergetics (COX6C). Their combined evaluation offers substantial clinical value for predicting and understanding brain damage in patients with impaired lymphatic drainage, enhancing translational relevance for clinical settings. Although the analysis, given the limited sample size, is an exploratory finding, it remains crucial to validate these results with larger sample sizes to ensure the robustness and reliability of proposed biomarker candidates.

### Clinical relevance of the impaired dcLN drainage model.

Investigations of glymphatic transport under conditions of lymphatic drainage impairment have been conducted to understand glymphatic-lymphatic crosstalk, and how the 2 systems affect neurodegenerative processes (6, 8, 9, 12, 14, 50). From a treatment perspective, if a disease affects the mLV, one therapeutic strategy could be to enhance the mLV network with VEGF-C (14, 51). However, if the cervical lymphatic nodes are dysfunctional and not draining properly, alternative treatment may include cervical lymph node transplants or lymphovenous anastomosis (52–54). In our model, we deliberately impaired drainage at the level of the dcLN, and, clinically, this model might relate to situations wherein cervical lymph nodes are functionally obstructed (clinical lymphatic flow disorder) or excised (e.g., as in cancer surgical therapy). Clinically, such diseases include: (a) Idiopathic Parkinson’s disease (55), (b) facial and neck lymphedema observed following neck lymphadenectomy or radiation for head and neck cancer (54, 56) and (c) Impairment of thoracic lymphatic duct drainage, as seen in central lymphatic flow disorder (CLFD), often manifesting as anasarca (52, 53). Of note, in patients with facial lymphedema, lympho-venous anastomosis (LVA) has been successfully implemented as a surgical treatment (54). Furthermore, in pediatric CLFD cases, interventions such as thoracic duct embolization or LVA are curative treatment options (57). To illustrate clinical examples, we included 2 cases of CLFD. Supplemental Figure 8 shows whole body dynamic contrast MRI lymphangiography (DCMRL) images from 2 patients with CLFD in which extracranial and neck lymphatic vasculature as well as cervical lymph nodes are documented via Gd-contrast injection into liver, mesentery, and the inguinal lymph nodes (52, 53, 58). CLFD is a rare condition in neonates that can arise from multiple disorders, including elevated central venous pressure (CVP) (52, 53, 58). In CLFD with increased CVP there is functional obstruction of the thoracic duct outlet where it empties into the left subclavian vein, which can result in retrograde lymph flow and lymphedema, including in the neck, scalp, oropharynx, and nasopharynx. The retrograde flow in CLFD shows the interconnected lymphatic vascular networks between the neck, nasopharynx, oropharynx, and scalp (Supplemental Figure 8), which may serve as extracranial aberrant drainage pathways.

In conclusion, our approach to studying physiological and biochemical profiles in a rat model of chronic dcLN impairment presents an important platform for better understanding the consequences of glymphatic-lymphatic uncoupling. The comprehensive investigation presented reveals the brain’s remarkable capacity to adapt to and compensate for lymphatic dysfunction through alternative drainage mechanisms and accelerated parenchymal clearance. These adaptations, while beneficial in striving to achieve fluid homeostasis, are not without consequences, as evidenced by the emergence of low-grade inflammation, BBB leakage, and signs of neurodegeneration. The intricate interplay between mechanical stress, inflammatory responses, and neurodegenerative processes highlighted in this study provides a crucial foundation for future research aimed at deciphering the molecular underpinnings of these relationships.

## Methods

### Sex as a biological variable

In this study we only used female rats due to rodent body-weight constraints imposed by the 9.4T MRI bore size. Further, ideal body weights for the DCE-MRI studies at the level of the neck using small radio-frequency surface coils is less than 400 g and prohibited including larger male rats. We note that a recent study reported that glymphatic influx is not dependent on biological sex (59).

### Animals and overall study design

Sprague Dawley (SD) female rats purchased from Charles River (Charles River Laboratories International Inc.) and allowed at least 1 week to acclimatize prior to experimentation. The rats were housed in an environment with controlled temperature, individually ventilated cages, and humidity, 12/12 hour light cycle from 7:00 AM to 7:00 PM and fed standard chow and water ad libitum. Our study was divided into different experiments as outlined in Supplemental Table 1.

### Deep cervical lymph node cauterization surgery

Animals were administered analgesic Ethiqa XR (Fidelis) (0.65 mg/kg IP) preoperatively, as well as meloxicam (1.5 mg/kg IP) preoperatively and every 24 hours for 48 hours postoperatively. Aseptic survival surgery was performed under isoflurane anesthesia (2%–3% in 1:1 O_2_: Air). The dcLN were cauterized to obstruct all lymphatic influx to the node. All rats underwent regular postoperative monitoring in accordance with federal guidelines. The rats were allowed to survive 1 month after the surgical procedure.

### ICP measurements

All rats underwent the procedure under anesthesia and a small CSF catheter was positioned in the cisterna magna (17, 60). For all experiments, ICP was measured with a precalibrated pressure probe (Millar’s SPR-1000 Mikro-Tip mouse pressure catheter) inserted into a water-filled 23 G stub adapter and sealed by Touhy Borst (16, 61, 62). For ICP recording, the 23 G stub adapter containing the calibrated pressure-sensitive probe was attached to a CSF catheter. Raw voltage data from the pressure probe were recorded at a frequency of 10 kHz over 5 minutes; the 60 seconds of data after ICP stabilized was analyzed. Analysis of mean ICP was performed using LabChart version 8.

### DCE-MRI

All rats underwent MRI under anesthesia and a small CSF catheter was positioned via the cisterna magna prior to scanning (17). DCE-MRI acquisitions were performed on a Bruker 9.4 T/16 magnet (Bruker BioSpin). The rats were imaged in supine position and anesthesia was maintained with a subcutaneous infusion of dexmedetomidine (approximately 0.009 mg/kg/hr) supplemented with low-dose, approximately1% isoflurane (16, 17). The body temperature was kept normal and respiratory rate was kept in the range of 45–55 beats per minute during scanning.

#### DCE-MRI at the neck.

A 2-cm planar surface receive radiofrequency (RF) coil (Bruker BioSpin) placed above the neck was used for RF signal reception and a volume transmit RF coil was used as RF signal transmitter. A series of 3D T1 weighted scans were acquired dynamically before and after contrast administration using a single flip angle spoiled gradient echo (SPGR) sequence: TR = 15 ms, TE = 4 ms, FA = 15°, Average = 1, FOV = 30 × 30 × 30 mm, Matrix = 150 × 150 × 150 the spatial resolution = 0.200 × 0.200 × 0.200 mm, acquisition time/scan = 5 minutes 38 seconds. After five baseline scans, 30 μL of 25 mM GadoSpin-P (Miltenyi Biotech GmBH) dissolved in sterile 0.9% NaCl was infused at a rate of 1.5 μL/minute for 20 minutes into CSF. Postcontrast T1-weighted scans were acquired for approximately 180 minutes.

#### Whole-brain DCE-MRI.

Whole-brain DCE-MRI was performed as previously described (16, 17),and 30 μL of 1:10 gadoteric acid (Gd-DOTA, DOTAREM, Guerbert LLC) diluted in sterile water was infused at a rate of 1.5 μL/minute into CSF during imaging.

#### MRI data analyses for deriving anatomical brain masks.

The 3D PDW images were corrected for intensity in homogeneity using the N4 bias field correction algorithm (63) and then segmented into tissue and CSF brain and ROI masks of individual brains. These masks were used for the urOMT analysis of glymphatic influx and clearance (details below). All the spatial segmentations were performed with the SPM12 (http://www.fil.ion.ucl.ac.uk/spm) software package platform, using our custom-made tissue probability maps (64). The brain parenchymal (grey and white matter) and CSF compartmental probability maps were thresholded at 0.5, yielding corresponding binary masks in template space. Binary masks of ROIs were created by spatially normalizing ROIs included in the publicly available Waxholm rat brain atlas package (65) onto our custom-made SD rat template.

### DCE-MRI image analysis

All the DCE-MRI data was corrected for motion, followed by intensity normalization, smoothing, and then voxel-wise percent signal change to baseline was calculated using SPM12 (https://www.fil.ion.ucl.ac.uk/spm/), as previously described (17, 20). The signal intensity normalization in the brain DCE-MRI datasets were done via the external fiducial marker, while, for the neck DCE-MRI data, the submandibular jaw muscles were used for signal intensity normalization. The DCE-MRI acquired at the neck are prone to be noisier and these images were therefore further denoised, and to denoise the images simultaneously, we applied a 4D denoising filter (66, 67). The denoised “percent signal change from baseline images” were then used for further analysis.

### Neck DCE-MRI image analysis

The time series of postcontrast T1-weighted images at the level of the neck were summed and used as an anatomical template to manually outline volumes of interests (VOI) using PMOD (PMOD, version 3.908, PMOD Technologies LLC). ROIs included the cervical lymph nodes and signal streams along the neck vasculature. The time series of DCE-MRI images were normalized to the peak CSF signal intensity, and the time signal curves (TSC) were extracted using PMOD software. The normalized TSC underwent noise cancellation via a 2-time-step moving average analysis (XLSTAT 2021.1.1, Addinsoft [2021]).

### urOMT analysis of DCE-MRI images

The mathematical model used in the present work to process the DCE-MRI brain data is called the “unbalanced regularized optimal mass transport” (urOMT) model (see Supplemental Material for further information).

The DCE-MRI brain images were first processed to obtain parametric images of percentage change from baseline, which are directly proportional to the concentration of Gd-based tracer (68) and therefore can be represented by the mass density in the urOMT formulation. The DCE-MRI brain series were fed into the urOMT algorithm (21), which was run repeatedly between 2 adjacent images. For all rats, the urOMT processing was performed with the same settings and parameters with and as a monotonically increasing series from 5,000 to 40,000. Fourteen volumetric images of 10-minutes time-averaged *r-flux* maps, which are denoted as in the urOMT formulation, were consequently returned. In each *r-flux* map, the local voxel values reflect the rate of tracer entering (when positive) or the rate of tracer exiting (when negative) in unit of percentage per minute. Voxels with a *r-flux* value larger than 0.001 were defined as an influx voxel, while a voxel with a *r-flux* value lower than –0.001 as a clearance voxel. The volumes (in mm^3^) and the corresponding volume fraction (volume in mm^3^ divided by the total region volume in mm^3^) of influx and clearance were extracted from the brain regions of interest (brainstem and cerebellum). Similarly, regional *net rates* were calculated by taking the spatial average of the *r-flux* values in a given brain region. By extracting the net rates and volume fractions at all 14 time points, we derived their corresponding time trajectories of influx and clearance. The *r-flux* time trajectories underwent noise cancellation via a 1-time-step moving average analysis (XLSTAT 2021.1.1, Addinsoft (2021). The mean rate from 30 → 110 minutes was calculated and uses as an influx net rate and the mean rate from 120 → 160 minutes was used as a clearance net rate (Supplemental Figure 1E).

### Tandem mass tag labeling for proteomics analysis of CSF

CSF samples were digested using the FASP protocol with 10 kDa MWCO filters (69). The eluted Peptides were labeled with the TMT10plex kit (Catalog 90111, Thermo Fisher Scientific) per the manufacturer’s instructions. Samples were analyzed on an Orbitrap Fusion mass spectrometer coupled to an Easy-nLC 1200 via nano-ESI. Data were acquired using Xcalibur v4.5.

### TMT qualitative and quantitative analysis

The analysis of MS/MS data derived from peptides labelled with TMT10plex isobaric reagents were performed using the built-in algorithm for TMT MS3 analysis and quantitative proteomics provided by PEAKS XPRO. Raw MS/MS files from TMT-labeled peptides were filtered, de novo sequenced, and assigned with protein ID using PEAKS XPro software by searching against the rat (*Rattus norvegicus*) SwissProt revised database (10,193 entries). Data were validated using the FDR method built in the in PEAKS XPRO. The Quantitative module (Q) of PEAKS XPRO was used to calculate the isobaric tag ratios for the validated peptides and generate the protein ratios. The “auto normalization” function from the PEAKSQ module was enabled (70).

### Proteomic profiling of Cerebrospinal fluid — DDA and DIA PASEF analysis

CSF samples from sham and dcLN cauterized rats were collected and analyzed using our optimized protocol (71) and analyzed on a timsTOF Pro2 mass spectrometer using diaPASEF. Peptides were separated over 65 minutes at 300 nL/minute using a reversed-phase C18 column. For spectral library generation, 20% of each digested sample was pooled and analyzed using timsTOF Pro2 in DDA PASEF mode. Raw files from DIA PASEF mode were deconvoluted with PEAKS ONLINE. The raw files were filtered, de novo sequenced, and assigned a protein ID using Peaks XPRO or ONLINE software (Bioinformatics Solutions), by searching against the RAT (*Rattus Norvegicus*) revised SwissProt/UniProt database (10,256 entries, April 2025). Data from the label-free quantitation was validated using the FDR method built in PEAKS XPRO and protein identifications were accepted if they could be identified with a confidence score (−10 LogP) > 15 for peptides and (−10 LogP) > 15 for proteins; a minimum of 1 or 2 unique peptide(s) per protein after filtering for less than 3.0% FDR for peptides and less than 3% FDR for proteins identifications. Data were preprocessed based on the comparison group, and significance testing was performed using the model. Proteins with a fold change greater than 2 and a *P* value less than 0.05 were identified as significantly different in expression levels. Although several proteins in the cohort exhibited high statistical significance, they failed to meet our significance threshold after correction for multiple comparisons (Benjamini-Hochberg FDR method). To identify potential biomarkers, multivariate exploratory analysis using partial least-square discrimination analysis (PLS-DA) was performed with MetaboAnalyst Software V6.

### Gene ontology, pathway enrichment, and protein analysis of label-free and TMT proteomics data

Networks, functional analyses, and pathway enrichment were performed using Ingenuity Pathway Analysis (IPA; Ingenuity Systems, Redwood City, CA, USA) on differentially abundant proteins from TMT data (Supplemental Table 1). Gene symbols and rescaled log_2_ TMT ratios were uploaded to IPA and mapped onto the Ingenuity Knowledge Base. Networks were algorithmically generated based on molecular connectivity. Canonical pathways were identified using right-tailed Fisher’s exact test (*P* < 0.05), with significance defined as −log(*P*) > 1.3.

### Lipidomics sample processing and metabolite (oxylipins) extraction

Prior to metabolomic analyses, CSF samples (20 μL) were diluted in 180 μL of ice-cold acetonitrile:methanol:water (50:30:20 v/v/v). Suspensions were vortexed for 30 minutes at 4°C, and then centrifuged for 10 minutes at 18,213*g*, and 4°C. Supernatants were isolated for mass spectrometry–based characterization.

### Ultra-high pressure liquid chromatography-mass spectrometry lipidomics

Oxylipin analysis employed a Vanquish UHPLC system (Thermo Fisher Scientific) coupled to a Q Exactive mass spectrometer (Thermo Fisher Scientific). Samples were resolved across a 2.1 x 100 mm, 1.7 μm particle size Acquity UPLC BEH column (Waters) using a 7 minute, reversed-phase gradient at a constant flowrate. Raw files were converted to mzXML using RawConverter. Run order of samples was randomized and technical replicates were injected after every 4 samples to assess quality control. The resultant files were processed with El-Maven (Elucidata) alongside the KEGG database for metabolite assignment and peak integration, as previously described (72–74).

### Enzyme linked immunosorbent assay (ELISA) for the validation of proteomic data

To assess inflammatory status in CSF from young and middle-aged rats, concentrations of interleukin-1β (IL-1β) and the DAMP molecule HMGB-1 were quantified. HMGB-1 was measured using a rat-specific ELISA kit (AssayGenie), and IL-1β using the Quantikine ELISA Kit (R&D Systems), following the manufacturers’ protocols.

### Clinical case descriptions

Patient 1 was a 4-month-old ex-premature 24-week gestation female with severe lymphatic conduction disorder due to chronic lung disease (CLD). She exhibited a history of cholestatic jaundice, anasarca, anemia of prematurity, mild pulmonary stenosis, cytomegalovirus (CMV) infection, and failure to thrive. At the time of imaging, the central venous pressure was 19 mmHg.

Patient 2 was a six-month-old ex-premature 26-week gestation and exhibited chronic respiratory failure due to CLD with tracheostomy and was mechanical ventilation dependent. In addition, the patient had a history of adrenal insufficiency, seizure disorder, congenital hypothyroidism, and dysregulated lymphatics with protein-losing enteropathy and was gastrostomy tube dependent.

During MRI imaging the patients were under general anesthesia. The MRI data were acquired on a 1.5T Siemens Avanto scanner.

The MRI images was acquired on a 1.5T Siemens Avanto scanner using a 3D spoiled gradient-recalled-echo T1-weighted image sequence with fat suppression acquired with a voxel size of 1.1 × 1.1 × 1.1 mm^3^. The images shown were acquired 8 minutes after contrast injection.

### Statistics

A formal a priori power and sample size calculation was not performed for this study due to the absence of pilot data in the rat directly relevant to our specific research question and design. Without reliable estimates of effect sizes, outcome variability, or event rates, any assumptions used for power estimation would be speculative and would risk drawing misleading conclusions about the adequacy of the sample size. Such analyses, which estimate the probability of detecting an observed effect given the sample size and variance, do not provide meaningful insights beyond what is already conveyed by confidence intervals and *P* values. In light of these constraints, our analysis focuses on effect estimates and their confidence intervals to assess the precision and potential clinical relevance of the findings. Data are presented as mean, SDs, and 95% CIs unless otherwise stated. Two-group comparisons were made using a 2-tailed independent *t* test assuming unequal variances (Welch’s *t* test). For analyzing the urOMT time trajectories of tissue volumes exhibiting influx and clearance, a linear mixed model with a heterogeneous variance covariance matrix for repeated measures with independent variables including group (sham vs dcLN cauterized groups), time and the time × group interaction was fit to compare the mean differences of different outcomes between groups. Group differences were calculated using a post hoc pairwise estimated marginal means with adjustments for multiple comparisons using the FDR correction. Statistical analyses were performed using IBM SPSS Statistics, Version 26, and GraphPad Prism software (version 10.1.2, LLC).

Separate statistical analyses of proteomics and metabolomics data were performed. Graphs and statistical analyses (either *t* test or ANOVA), multivariate analyses, including Partial Least Squares-Discriminant Analysis (PLS- DA), and metabolite pathway enrichment analysis were performed using MetaboAnalyst 5.0. The PEAKS built-in *t* test /ANOVA (significance for *P* < 0.05) were used to assess the statistical significance of the fold changes in the protein expression. Networks, functional analyses, and biochemical and cellular pathways were generated by the ingenuity pathway analysis (IPA; Ingenuity Systems). The calculated FCs for each sample corresponding to sham and cauterized groups were imported in IPA for generating the networks and assessing the pathways activation *z* scores (where *z* < –2.0 is inhibition and *z* > 2.0 is activation). A right-tailed Fisher’s exact test with Benjamini-Hochberg correction was used to calculate *P* values (significance level was set at *P* < 0.05). The IPA analysis identified the pathways from the IPA library of canonical pathways that were most significant to the dataset (−log (*P* value) > 1.3). Graph plotting and statistical analysis was performed using Windows GraphPad Prism 9 (GraphPad Software). Numerical results are reported as mean ± SEM or ± SD when appropriate. A comparison between more than 2 groups was performed using 2-tailed unpaired 1-way ANOVA or 2-way ANOVA followed by multiple comparison tests and Holm–Šidák method. Results were considered statistically significant if *P* ≤ 0.05. All statistics are reported in the figure legends.

### Study approval

All the animal work was approved by the local IACUC at Yale University, New Haven, Connecticut, USA. The 2 clinical cases shown comply with the HIPAA Privacy Rule and accordingly the MRI images presented are deidentified of protected health information.

### Data availability

All data supporting the findings of this study are available within the article and its Supplemental Materials. The mass spectrometry proteomics data have been deposited to the ProteomeXchange Consortium via the PRIDE partner repository under the dataset identifiers PXD063161 and PXD063564.

## Author contributions

ZG, AB, and JCM performed all dcLN cauterization and sham surgeries, performed all intracranial pressure experiments, analyzed, and interpreted the data. ZNK performed all omics analyses and interpreted the data with LS. SK performed all of the MRI experiments and data analysis was performed by SK, HL, and HB. The urOMT analysis was performed by KX and XC, and AT and wrote the corresponding method section. HML and HB performed the statistical analysis. JK and XG provided intellectual contribution and helped interpret the data. LS oversaw the omics data analysis and contributed to the manuscript writing. YD provided the 2 clinical cases. HB conceived the study, oversaw data analysis and wrote the manuscript. ZG and ZNK share first coauthorship and had distinct but equally substantial roles within the project. ZHG focused on the experimental animal work while ZNK was responsible for the omics data analysis.

## Supplementary Material

Supplemental data

Supplemental table 2

Supplemental table 3

Supporting data values

## Version 1 07/15/2025

In-Press Preview

## Version 2 09/02/2025

Electronic publication
