# Supplementary material for: Age-dependent brain responses to mechanical stress determine resilience in a chronic lymphatic drainage impairment model
Source: J Clin Invest. 2025 Jul 15;135(17):e182555. doi: 10.1172/JCI182555 (PMC12404751; doi:10.1172/JCI182555)
Supplement: Supplemental data [file jci-135-182555-s126.pdf]

Supplemental Material

Age-dependent brain responses to mechanical stress determine resilience in a chronic lymphatic drainage impairment model

Zachary Gursky, Zohaib Nisar Khan et al.

Supplemental Figures 1-8.

Supplemental Methods.

Supplemental Table 1.

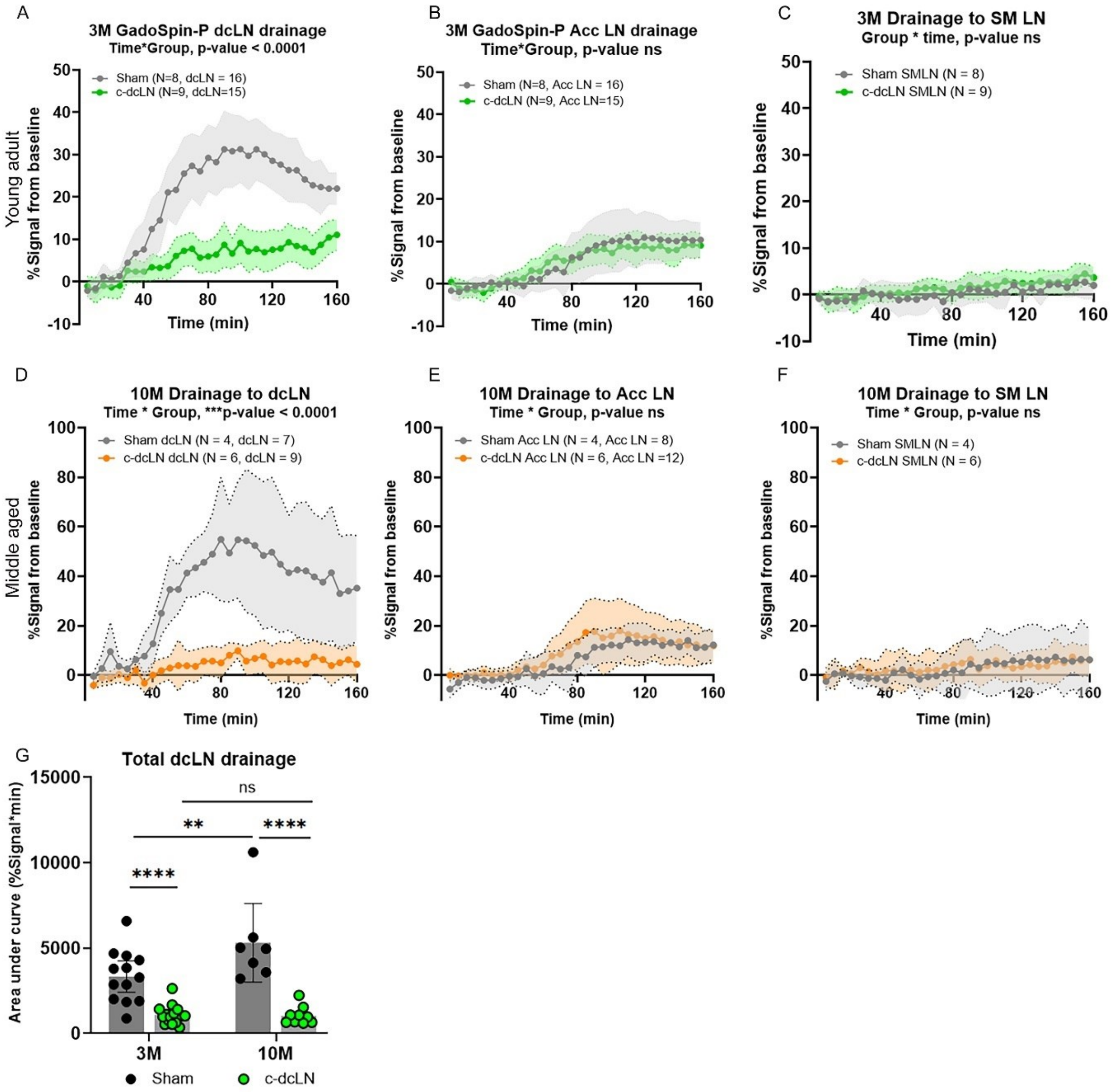

Supplemental Figure 1: Cervical lymph node drainage status across groups

(A-C) Graphs of the time signal curves of GadoSpin-P drainage from the brain to the deep cervical lymph nodes (dcLN) (A), accessory lymph nodes (Acc LN) (B) and submandibular lymph nodes (SM LN) from the 3-month-old sham and dcLN cauterized rat. (D-F) Data are presented as the mean and 95% confidence intervals. G) Graph of the total drainage – measured as area under curve - to the dcLN across groups. A linear mixed model for repeated measures (LMM-RM) with False Discovery Rate for multiple comparisons was utilized to assess differences across groups. The time*group interaction effects are listed for each graph. **p<0.01, ****p<0.0001, ns=non-significant.

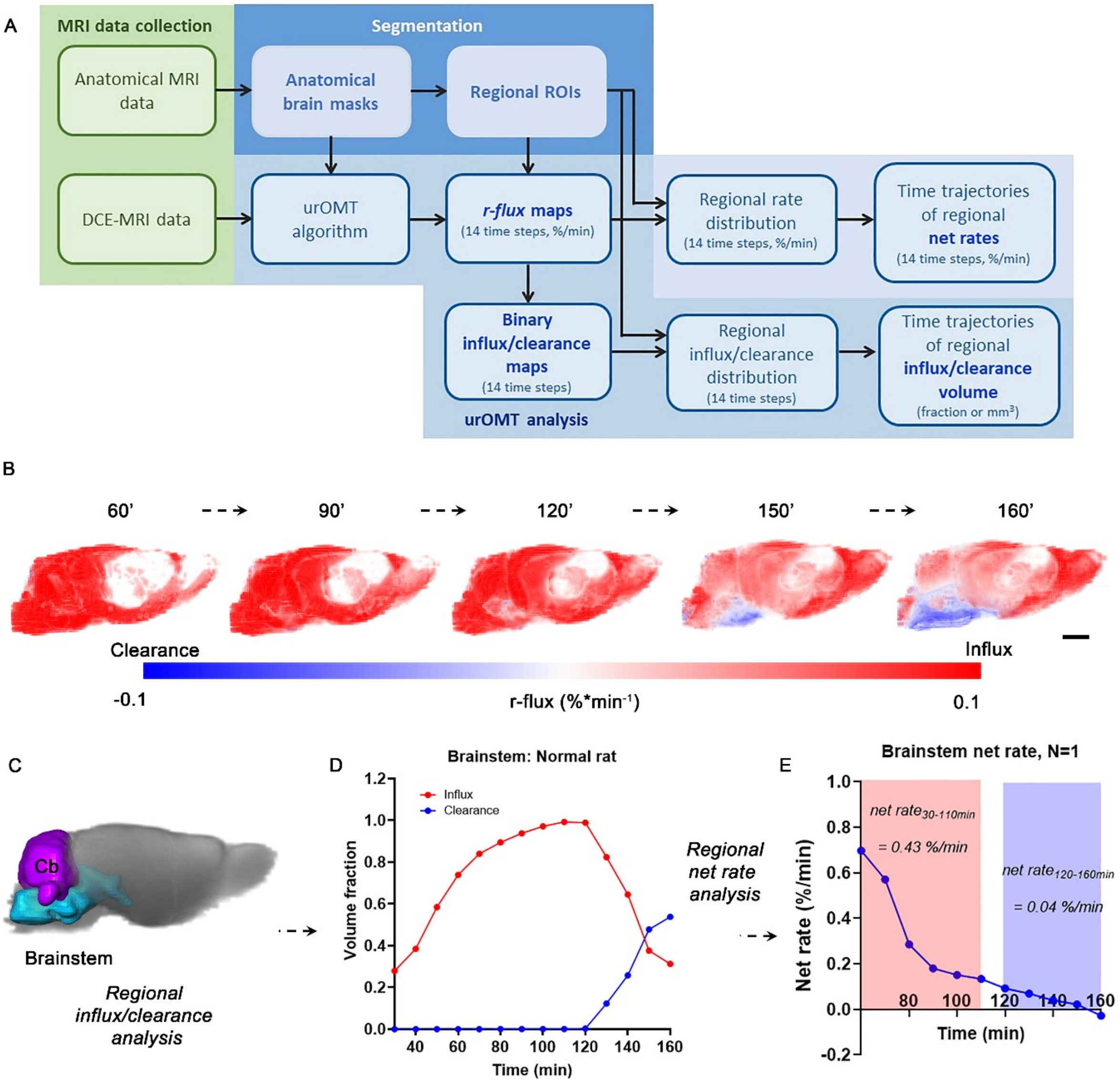

Supplemental Figure 2: Processing pipeline for urOMT analysis

(A) Overview of the urOMT computational processing pipeline of DCE-MRI data. (B) A representative time series of *r-flux* brain maps from a 3-month-old sham rat (this example is also shown in Figure 3A). Positive *r-flux* values in red color tones denote glymphatic influx and negative *r-flux* values in blue indicate clearance. Scale bar = 3 mm. (C) 3D anatomical rat brain with brain regions of interest outlined. Cb=cerebellum. (D) Graphs showing the time-trajectories of influx and clearance volume fractions in the brainstem. (E). Corresponding time-course of net rate changes in the brainstem from a sham rat. The red- and blue colored areas represent influx- and clearance dominated phases, respectively.

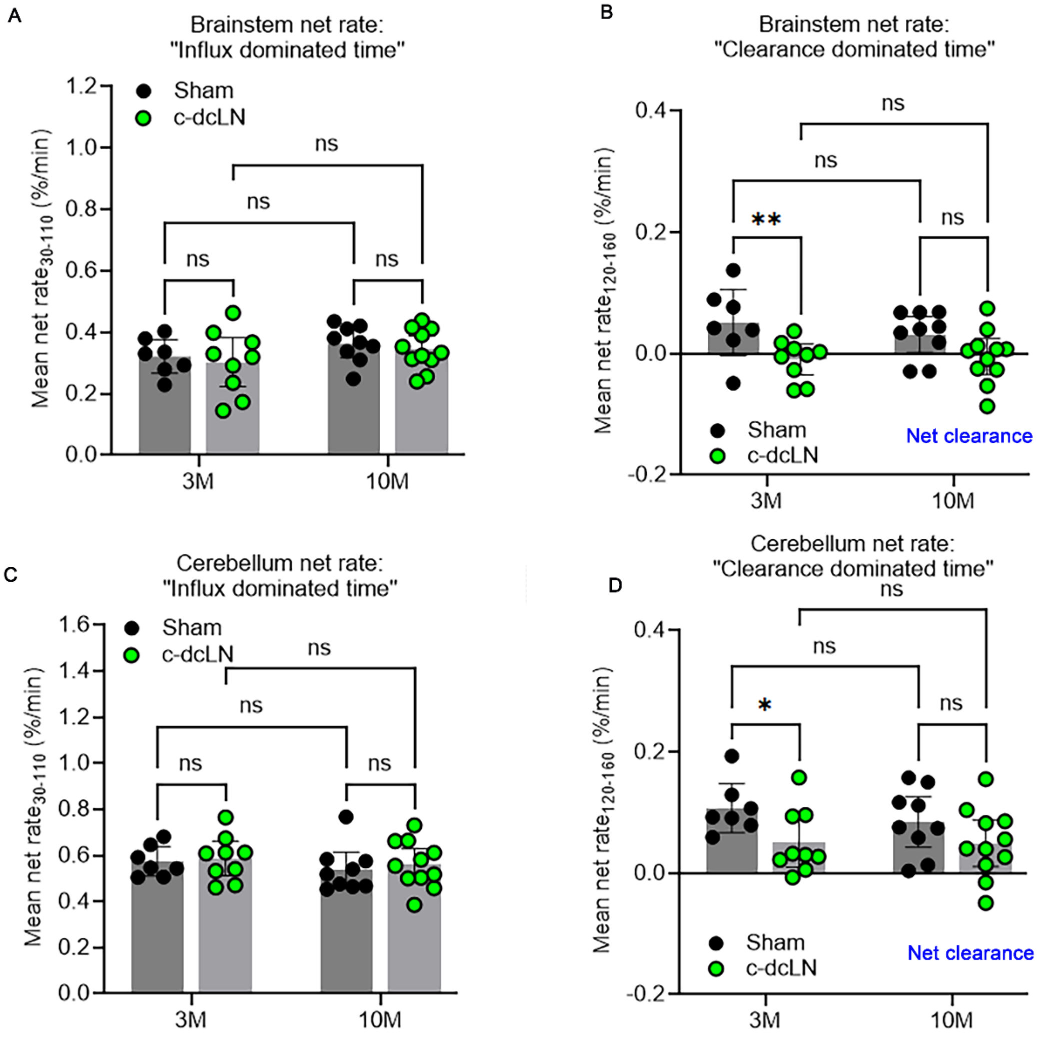

Supplemental Figure 3: Mean net rates of influx and clearance predominated transport across groups.
(A) Graph of the mean net rates in the brainstem during the influx dominated phase (from 30min → 110 min) of sham and rats exposed to dcLN cauterization (c-dcLN) across 3 months (M) and 10M cohorts. (B) Graphs of the mean net rates in the brain stem during the clearance predominated phase (from 120min → 160 min) from sham and c-dcLN rats across age cohorts. (C, D) Corresponding graphs from the cerebellum across the groups. Data are mean and 95% confidence intervals. A linear mixed model for repeated measures (LMM-RM) with False Discovery Rate correction for multiple comparisons was utilized to assess differences across groups. **p-value < 0.01; *p-value < 0.05.

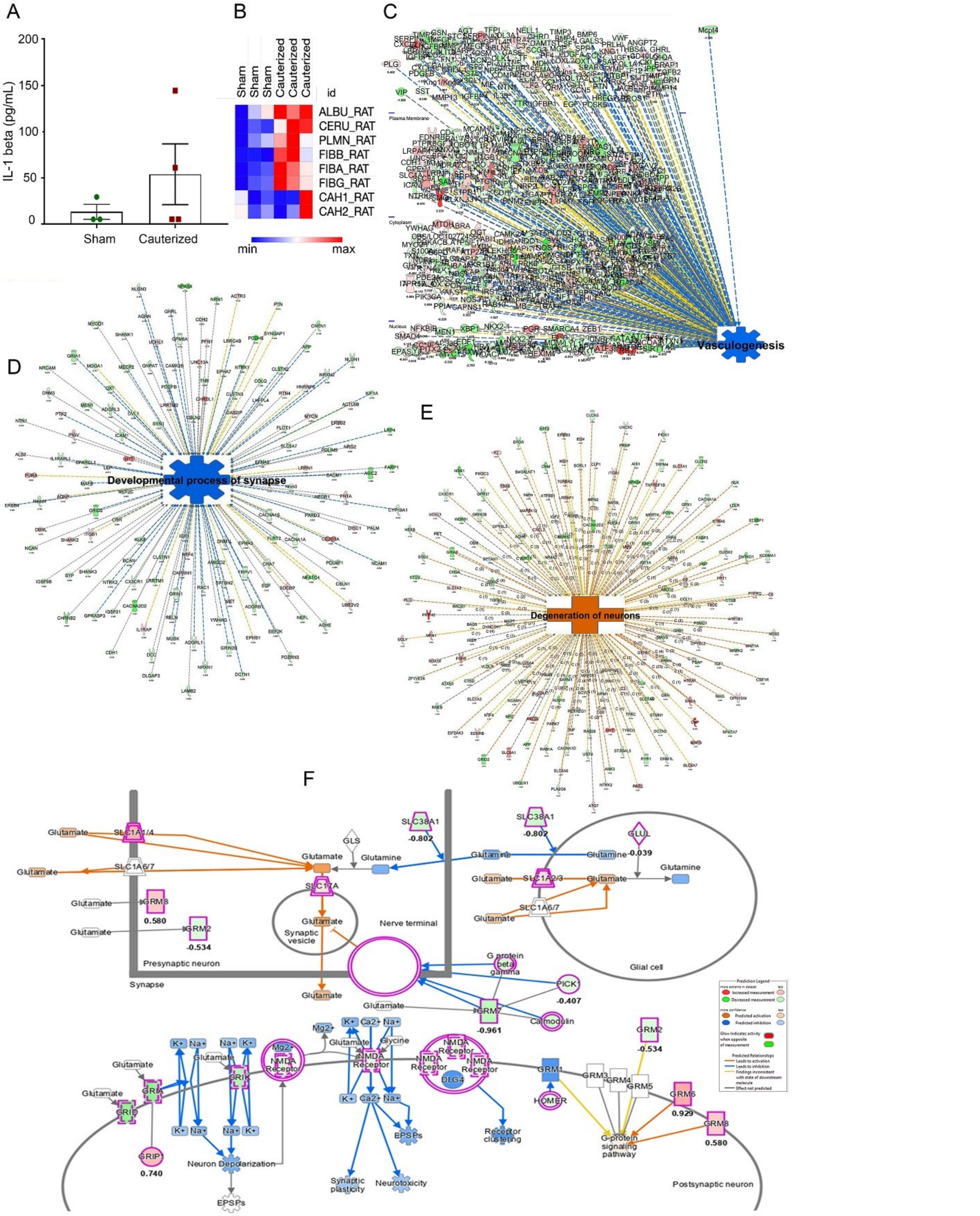

Supplemental Figure 4: Impaired brain lymphatic drainage induces a proteomic signature of disrupted synaptic activity, neurodegeneration and inflammation in young rats.

(A) Circulating levels of IL-1β in the CSF of dcLN and sham rats as detected by ELISA. Each data point corresponds to one biological replicate, data are reported as means ± SEM (p-value = 0.543). (B) Heatmap of the relative protein abundances, represented as normalized TMT reporter intensities, mapped in the CSF of dcLN and sham groups. (C, D, E) IPA predicted inhibited/downregulated and activated/upregulated pathways in the CSF proteome of dcLN-cauterized rats vs sham control. The identified proteins are represented as gene symbols and predicted to be up- (in red) or down- regulated (in green). For network generation, datasets containing gene symbols were uploaded into the IPA application together with the rescaled log2 transformation of the average protein's area ratios corresponding to the dcLN cauterization/sham dataset reporting significant changes (p-value < 0.05, by ANOVA/t-tests). Proteins and pathways are reported in Supplemental Table S3. (F) IPA-generated glutamate receptor signaling pathway predicted to be inhibited/downregulated in dcLN-cauterization group as compared to sham controls. Green depicts proteins down-regulated, whereas red colors depict proteins up-regulated in the CSF dataset. The color intensity directly correlates with fold changes, expressed as log2 ratio (MS3 input-values).

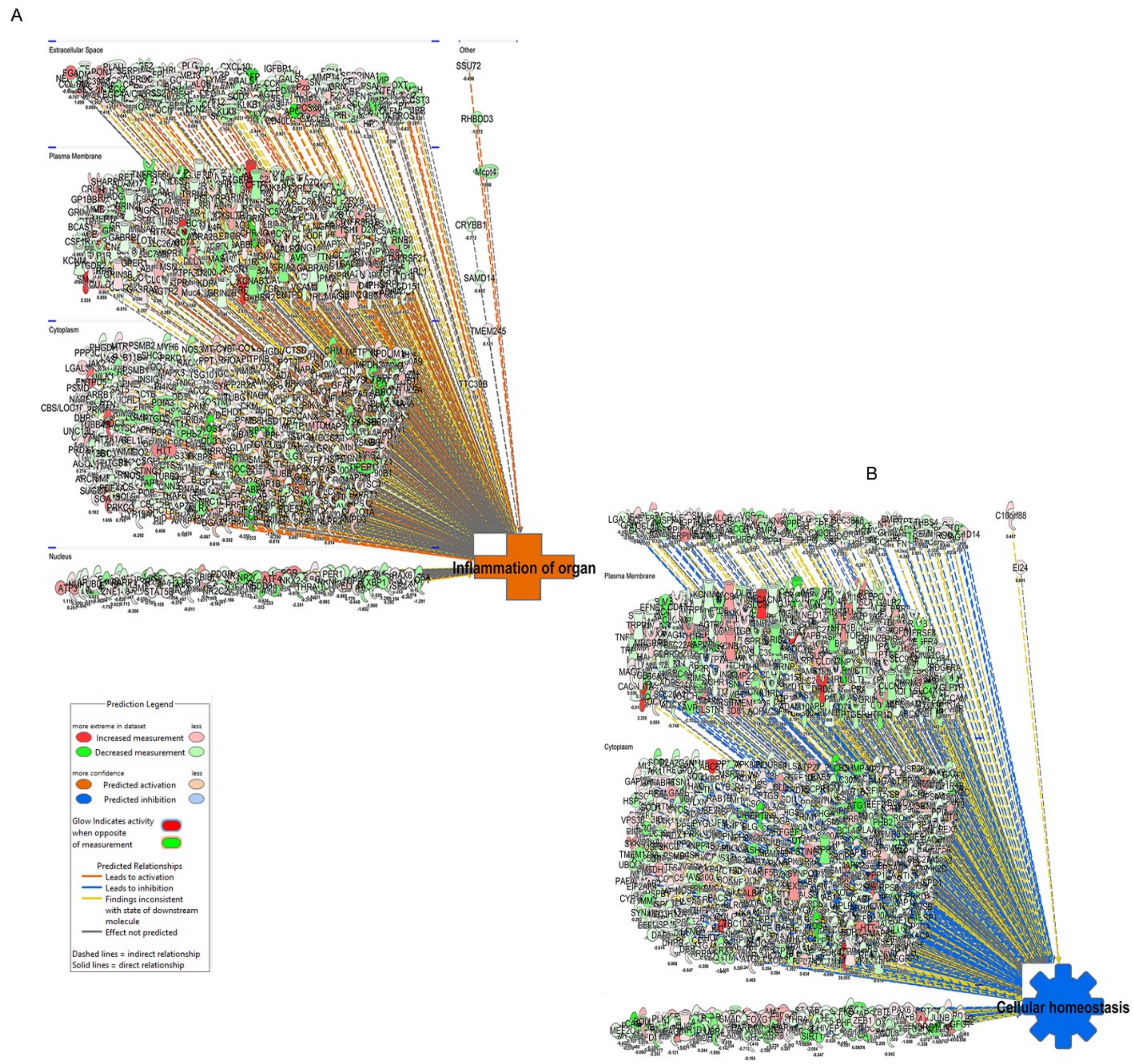

Supplemental Figure 5: Impaired brain lymphatic drainage induces a proteomic signature of compromised cellular homeostasis in young rats.

(A, B) Quantitative IPA analysis predicted the activation of inflammatory pathways and compromised cellular homeostasis. Identified proteins are represented as gene symbols and are predicted to be up-regulated (in red) or down-regulated (in green) in the comparison between dc-LN and sham groups. For network generation, datasets containing gene symbols were uploaded into the IPA application along with their rescaled log2 transformation of average protein area ratios. Proteins with significant changes ($p<0.05$, by ANOVA/t-tests) are reported in Supplemental Table 2.

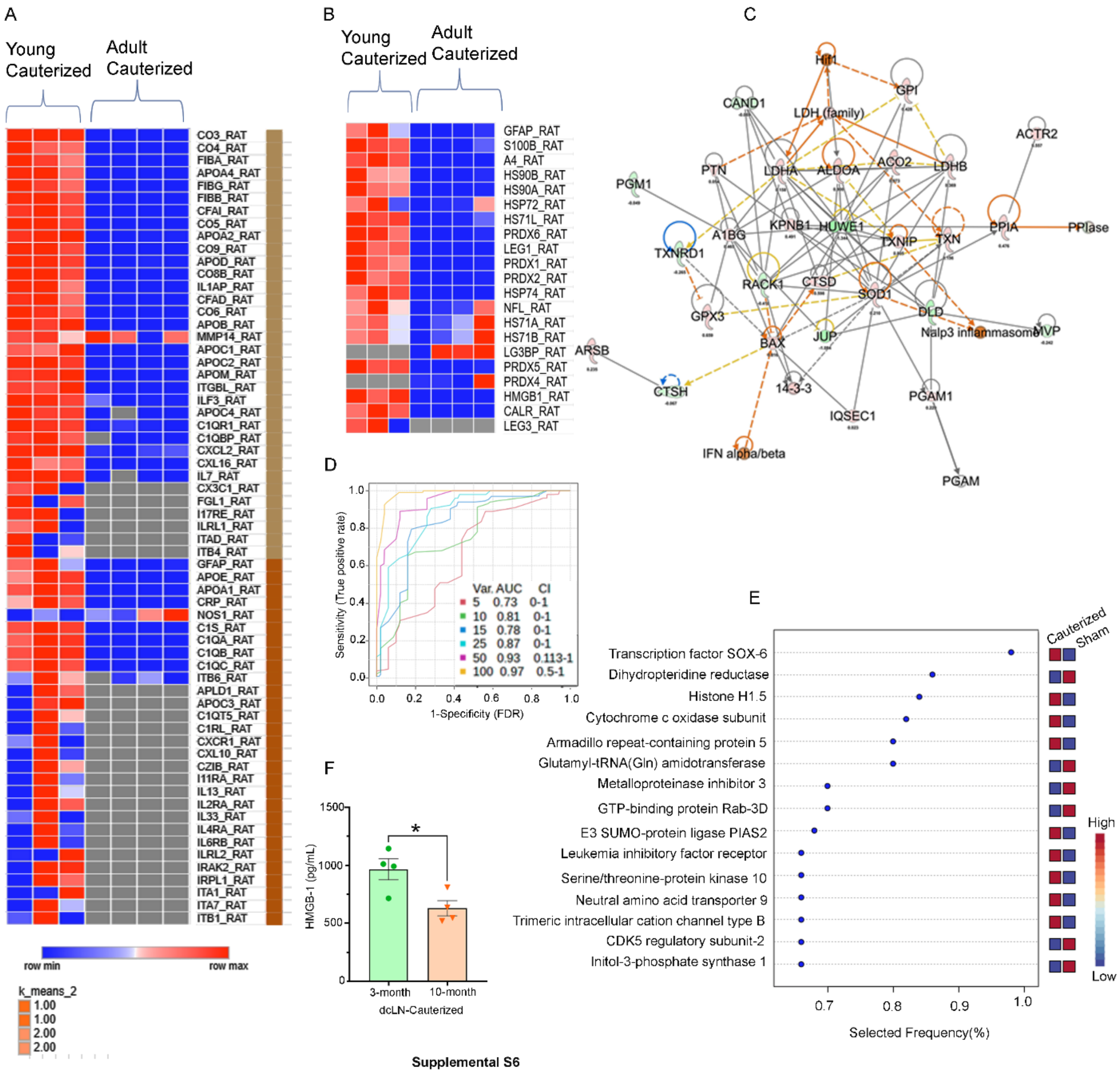

Supplemental S6

Supplemental Figure 6: Impaired brain lymphatic drainage induces a differential proteomic signature in young and old dc-LN cauterized rats.

(A, B) Pearson correlation heatmap of proteins associated with inflammation, cellular damage, and damage-associated molecular patterns (DAMPs) identified in all biological replicates of dc-LN cauterized rats from young and old cohorts. The heatmap highlights significant differences in the inflammatory and DAMP-associated proteomic abundance between the two cohorts. Blue indicates low differential protein abundance, red indicates high differential protein abundance, and grey indicates the absence of the protein. The list of proteins is reported in Supplemental Table 2. (C) Schematic representation of the highest-scoring network pathways depicting the involvement of differentially regulated proteins in processes related to cellular stress and survival,

free radical scavenging, and organismal injury and abnormalities. Nodes colored blue represents down-regulation, and those colored orange represent up-regulation. (D) Multivariate receiver operating characteristic (ROC) analysis, showing the feature numbers the AUCs and the confidence intervals of the six models. (E) Variable importance in projection (VIP) plots showing the top 15 potential biomarkers, indicating the most discriminating protein candidates in descending order of importance. The horizontal axis shows their corresponding percentage selected frequency for generating the ROC curves in the 9-month cohort. (F) CSF presence of DAMPs (HMGB-1) is more abundant in the young as compared to the 10M middle-aged rats. Comparative analysis between 3-Month and 9-Month cohorts (n = 4/group).

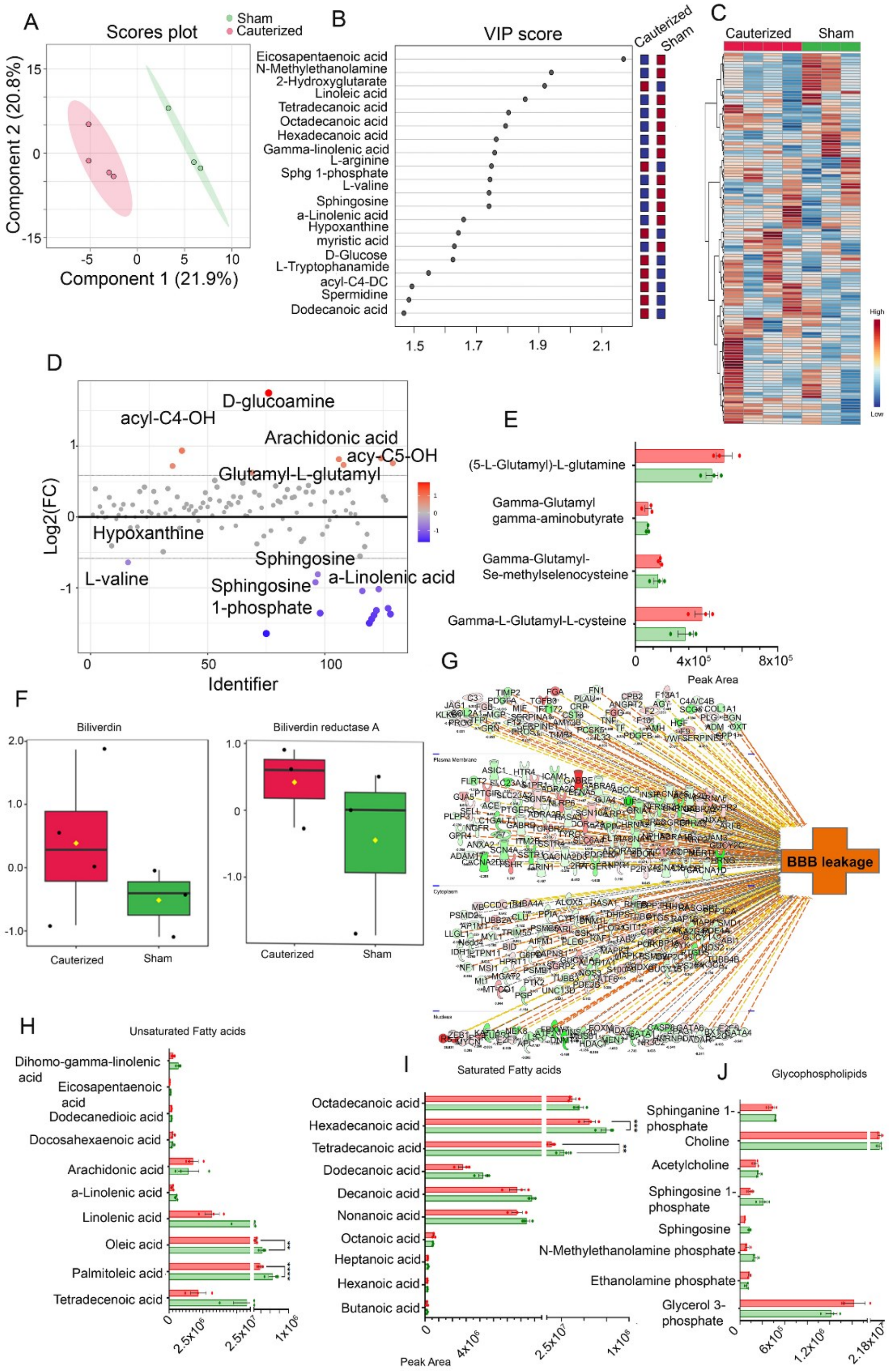

Supplemental Figure 7: Impaired lymphatic brain drainage is associated with metabolic reprogramming and accumulation of biomolecules related to inflammation and neurodegeneration in young rats.

(A) 2D PLS-DA score plot based on targeted metabolomic profile of CSF obtained from sham (green) and dcLN-cauterized (red) subjects highlighting the separation between two groups with ellipses representing the 95% confidence intervals of the group clustering. (B) Associated Variable Importance in Projection (VIP) scores ranking the most important metabolites (VIP > 1) that contribute to the separation in the PC 1 dimension of the two groups. The colored boxes on the right-hand side of the VIP score plot denote relative metabolite concentrations between the two groups. (C) HCA heat map generated by Metaboanalyst (using Euclidean clustering, Ward method) showing the deregulated profile of metabolites in the sham and dcLN-cauterized groups. Each colored cell on the map corresponds to a normalized concentration value, with samples in columns and compounds in rows. Red and green colors indicate positive and negative correlations, respectively. (D) Deregulated pattern of quantified metabolites in the comparison groups (dcLN cauterization/sham) (E) Boxplot of the relative concentration of significantly altered metabolites ($p<0.05$). Y axes are represented as relative units. Due to normalization process we obtained negative scale of Y-axis in some of the bins (Metaboanalyst program analysis). Each point represents a single animal. Statistical significance was determined using a two-way ANOVA (or mixed model). All error bars represent SEM. * $p<0.05$, ** $p<0.01$, *** $p<0.001$. (F) Boxplot of relative concentrations of significantly altered metabolites ($p<0.05$). Y axes are represented as relative units. (G) IPA-generated pathway showing activation/upregulation of blood-brain-barrier disruption, and the associated macromolecular complexes affected by impaired dcLN drainage in the CSF analyzed by MS3-TMT proteomics. Green depicts proteins down-regulated, whereas red colors depict proteins up-regulated. The color intensity directly correlates with fold changes, expressed as log2 ratio (Input-values). (H, I, J) Lipidomic analyses revealed changes in fatty acids (saturated, unsaturated, glycerophospholipids) in CSF of dcLN-cauterized compared to the sham rats. Each point represents a single animal. Statistical significance was determined using a two-way ANOVA (or mixed model). All error bars represent SEM. * $p<0.05$, ** $p<0.01$, *** $p<0.001$.

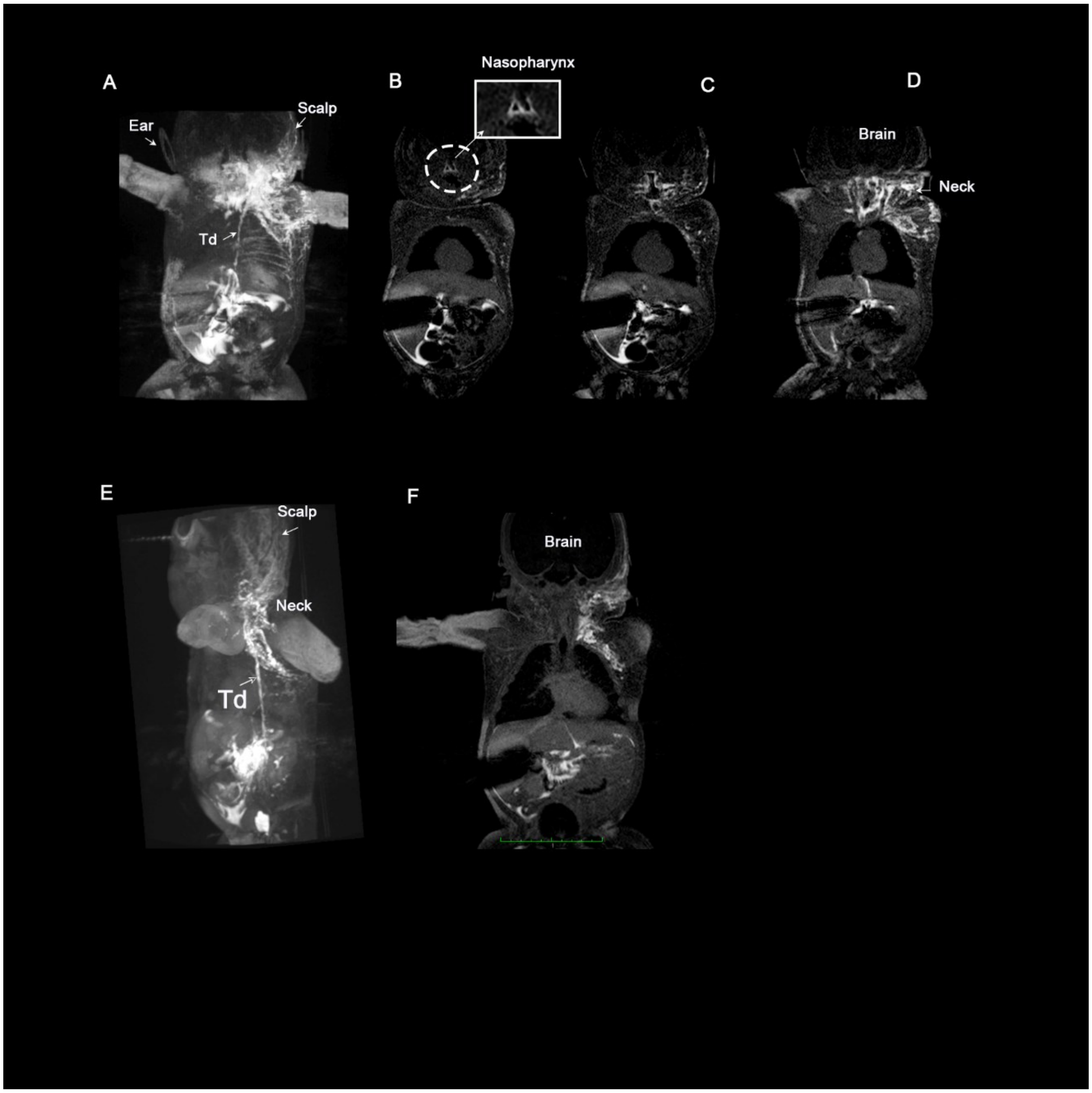

Supplemental Figure 8: Central lymphatic outflow disorder in two patients revealing interconnected lymphatic networks in neck, nasopharynx, oropharynx, and scalp.

Dynamic contrast enhanced magnetic resonance lymphangiography (DCMRL) of two patients (neonates). (A-D) DCMRL of Patient 1 shows evidence of chronic lymphatic flow disorder by demonstration of dilated thoracic duct (Td) and dense neck lymphatic vasculature connecting with multiple lymphatic networks in the neck and nasopharynx. (E-F) Patient 2's DCMRL reveals a dilated Td and extensive lymphatics at the neck interconnecting with lymphatics in the scalp.

Methods

The mathematical unbalanced optimal mass transport model.

The urOMT model works by finding the optimal transport strategy from one mass density distribution to another, during which the transport process is governed by the widely used advection-diffusion equation in fluid dynamics. Mathematically, the urOMT model can be formulated as follows. Suppose one is given an initial mass density function $\rho_0(x) \geq 0$ and a final one $\rho_1(x) \geq 0$ in a region $\Omega \subseteq \mathbb{R}^3$; the urOMT model solves

$$\min_{\rho,v,r} \int_0^1 \int_\Omega \rho(t,x)(\parallel v(t,x) \parallel^2 + \alpha r(t,x)^2)\, dx\, dt$$

subject to

$$\begin{cases} \dfrac{\partial \rho}{\partial t} + \nabla \cdot (\rho v) = \sigma \Delta \rho + \rho r, & (2) \\ \rho(0,x) = \rho_0(x), \quad \rho(1,x) = \rho_1(x). & \end{cases}$$

In the above, $\rho(t,x): [0,1] \times \Omega \to \mathbb{R}^+$ is the time-dependent mass density function that can represent the concentration of a quantity; $v(t,x): [0,1] \times \Omega \to \mathbb{R}^3$ is the time-dependent velocity field that describes the directional flow of a quantity; $r(t,x): [0,1] \times \Omega \to \mathbb{R}$ is called the relative source which is the rate of ingress (when $r > 0$) and egress (when $r < 0$) at the quantity into and out of the system; $\sigma > 0$ is the constant diffusion coefficient; $\alpha > 0$ is the weighting parameter of the source term in the cost function. Equation (2) is the unbalanced version of advection-diffusion equation.

Supplemental Table 1: Overview of Experimental Cohorts studied

Cohorts	Study objectives	CSF Gd-tracer	CSF analysis
3M, ♀ Sham (N=8) and c-dcLN (N=9) rats	Drainage from the CSF/brain → cervical lymph nodes	GadoSpin-P (MW 200kDa)	No
3M, ♀ Sham (N=7) and c-dcLN (N=9) rats	To measure brain glymphatic influx and clearance	Gadoteric acid (Gd-DOTA, (MW 559 Da)	Yes
3M, ♀ Sham (N=8) and c-dcLN (N=11) rats	To measure intracranial pressure (ICP) and physiological variables	N/A	Yes
10M, ♀ Sham (N=4) and c-dcLN (N=5) rats	Drainage from the CSF/brain → cervical lymph nodes	GadoSpin-P (MW 200kDa)	No
10M, ♀ Sham (N=9) and c-dcLN (N=11) rats	To measure brain glymphatic influx and clearance	Gadoteric acid (Gd-DOTA, (MW 559 Da)	Yes
10M, ♀ Sham (N=3) and c-dcLN (N=5) rats	To measure intracranial pressure (ICP) and physiological variables	N/A	No
M = months, SD = Sprague Dawley rats, c-dcLN = cauterization of deep cervical lymph nodes. MW = molecular weight.			
